# Tumor‐Associated Sympathetic Nerves Promote the Progression of Epstein‐Barr Virus‐Positive Diffuse Large B‐Cell Lymphoma

**DOI:** 10.1002/advs.202413580

**Published:** 2025-06-09

**Authors:** Silan Huang, Dexin Lei, Linbin Yang, Aiwei Bi, Yanlou Wang, Peng Zhang, Dongyu Zhuang, Honglian Liu, Qingqing Cai, Man Nie, Yi Xia

**Affiliations:** ^1^ Department of Medical Oncology State Key Laboratory of Oncology in South China Guangdong Provincial Clinical Research Center for Cancer Collaborative Innovation Center for Cancer Medicine Sun Yat‐sen University Cancer Center Guangzhou 510060 P. R. China; ^2^ Breast Tumor Center Guangdong Provincial Key Laboratory of Malignant Tumor Epigenetics and Gene Regulation Medical Research Center Sun Yat‐sen Memorial Hospital Sun Yat‐sen University Guangzhou Guangdong P. R. China; ^3^ Biotherapy Center Sun Yat‐sen Memorial Hospital Sun Yat‐sen University Guangzhou 510120 P. R. China

**Keywords:** EBV‐positive diffuse large B‐cell lymphoma, progression, sympathetic nerve, β2‐adrenergic receptors

## Abstract

Epstein‐Barr virus‐positive (EBV^+^) diffuse large B‐cell lymphoma (DLBCL) exhibits a poorer prognosis with limited treatment options. Although recent evidence indicates that the peripheral nervous system is associated with tumor progression, its role in EBV^+^DLBCL remains poorly understood. In the cohort, patients with EBV⁺DLBCL exhibit significantly shorter overall survival (OS). Two EBV^+^DLBCL cell lines are established and characterized. Although cell proliferation does not differ significantly in vitro, tumors derived from EBV^+^ DLBCL cells demonstrate accelerated growth compared to their parental counterparts in mouse models. Mechanistically, transcriptome analysis reveals the upregulation of axonogenesis‐related genes and pathways in EBV^+^DLBCL tumors. Immunostaining confirms increased nerve fiber infiltration in SUDHL6‐EBV xenografts and enhance neurite outgrowth from dorsal root ganglia co‐cultured with EBV^+^DLBCL cells. Both in vitro and in vivo experiments show that sympathetic nerves promote tumor growth via β2‐adrenergic receptors (β2ARs), which are attenuated by selective β2AR blockers. Clinically, EBV⁺DLBCL patient samples show more sympathetic nerve fibers and higher β2AR expression, both of which are associated with poorer survival. Furthermore, a meta‐analysis suggests that beta‐blocker use is linked to a reduced risk of cancer‐specific mortality. Together, these findings suggest that sympathetic nerve innervation drives the progression of EBV^+^DLBCL via β2ARs, highlighting a potential therapeutic target.

## Graphical abstract

Sympathetic nerve innervation promotes the progression of Epstein‐Barr virus‐positive diffuse large B‐cell lymphoma (EBV^+^ DLBCL) via β2‐adrenergic receptors. The discovery of enhanced nerve infiltration and β2AR expression reveals a novel mechanism and highlights the sympathetic–β2AR axis as a promising target for therapeutic intervention in EBV^+^ DLBCL.

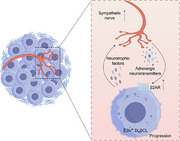

## 1 Introduction

Epstein‐Barr virus‐positive (EBV^+^) diffuse large B‐cell lymphoma (DLBCL) is a distinct entity recognized by the World Health Organization (WHO) since 2016, for which there is currently no established standard‐of‐care therapy.^[^ ^1^, ^2^, ^3^ ^]^ Most patients are treated with the same regimens used for newly diagnosed DLBCL, not otherwise specified (NOS)^[^ ^4^, ^5^ ^]^, yet they typically experience poorer outcomes compared to those with DLBCL, NOS^[^ ^6^, ^7^, ^8^ ^]^, underscoring the urgent need to elucidate the underlying mechanisms and develop novel treatment strategies. Most studies have implicated EBV‐driven oncogenic mechanisms, including the activation of the NF‐κB pathway and upregulation of BCL2^[^ ^9^, ^10^ ^]^ in tumor cells, alongside tumor environment (TME) modulation, such as M2‐type tumor‐associated macrophages (TAMs)^[^ ^11^ ^]^ polarization and PD‐L1 upregulation.^[^ ^12^ ^]^ However, few studies have focused on the role of the peripheral nervous system, which also plays a crucial role in cancer development and progression.

Peripheral nerves are present in nearly all tissues and participate in maintaining tissue homeostasis, regulating immune responses, and promoting tissue repair. Emerging evidence reveals that tumors actively recruit nerve fibers into their microenvironment by releasing neurotrophic factors^[^ ^13^, ^14^, ^15^ ^]^ and axon guidance molecules.^[^ ^16^ ^]^ Strikingly, different nerve subtypes exert paradoxical effects on cancer progression: while sympathetic (adrenergic) signaling promotes the development of prostate^[^ ^17^ ^]^ and pancreatic cancers^[^ ^15^, ^18^ ^]^, it suppresses hematopoietic stem and progenitor cells.^[^ ^19^, ^20^ ^]^ Conversely, parasympathetic (cholinergic) signaling inhibits pancreatic cancer^[^ ^21^ ^]^ but accelerates gastric^[^ ^14^, ^22^ ^]^ and prostate^[^ ^17^ ^]^ cancers. Sensory nerves accelerate the progression of head and neck cancers.^[^ ^23^ ^]^ Therapeutic strategies targeting nerve‐tumor crosstalk (e.g., via NGF inhibition) show promising anti‐tumor effects.^[^ ^24^, ^25^ ^]^ Clinically, increased nerve density is associated with poor prognosis in various cancers.^[^ ^26^, ^27^, ^28^ ^]^ Notably, while normal lymph nodes are densely innervated by sympathetic nerves that regulate immune function through β2‐adrenergic receptor signaling^[^ ^29^, ^30^, ^31^, ^32^ ^]^, the potential involvement of peripheral nerves in EBV^+^ DLBCL remains completely unexplored. This represents a critical knowledge gap, especially considering the aggressive clinical behavior of this lymphoma subtype and its distinctive tumor microenvironment characteristics.

Thus, this study aimed to explore the role of nerve innervation in EBV^+^ DLBCL. Here, we successfully established EBV^+^ DLBCL cell lines that exhibited microenvironment‐dependent accelerated in vivo growth. Further analysis showed increased infiltration of sympathetic nerves in EBV^+^ DLBCL tumors, and treatment with β2AR blockers effectively inhibited tumor progression. Clinically, increased sympathetic nerve infiltration and higher β2AR expression were found in EBV^+^ DLBCL patients and were linked to worse outcomes. Additionally, the use of beta‐blockers was associated with reduced cancer‐specific mortality. These results demonstrate that sympathetic nerves promote EBV^+^ DLBCL via β2AR signaling, revealing a novel therapeutic target.

## 2 Results

### 2.1 EBV^+^ DLBCL Patients Exhibit an Inferior Survival

**Figure 1:**
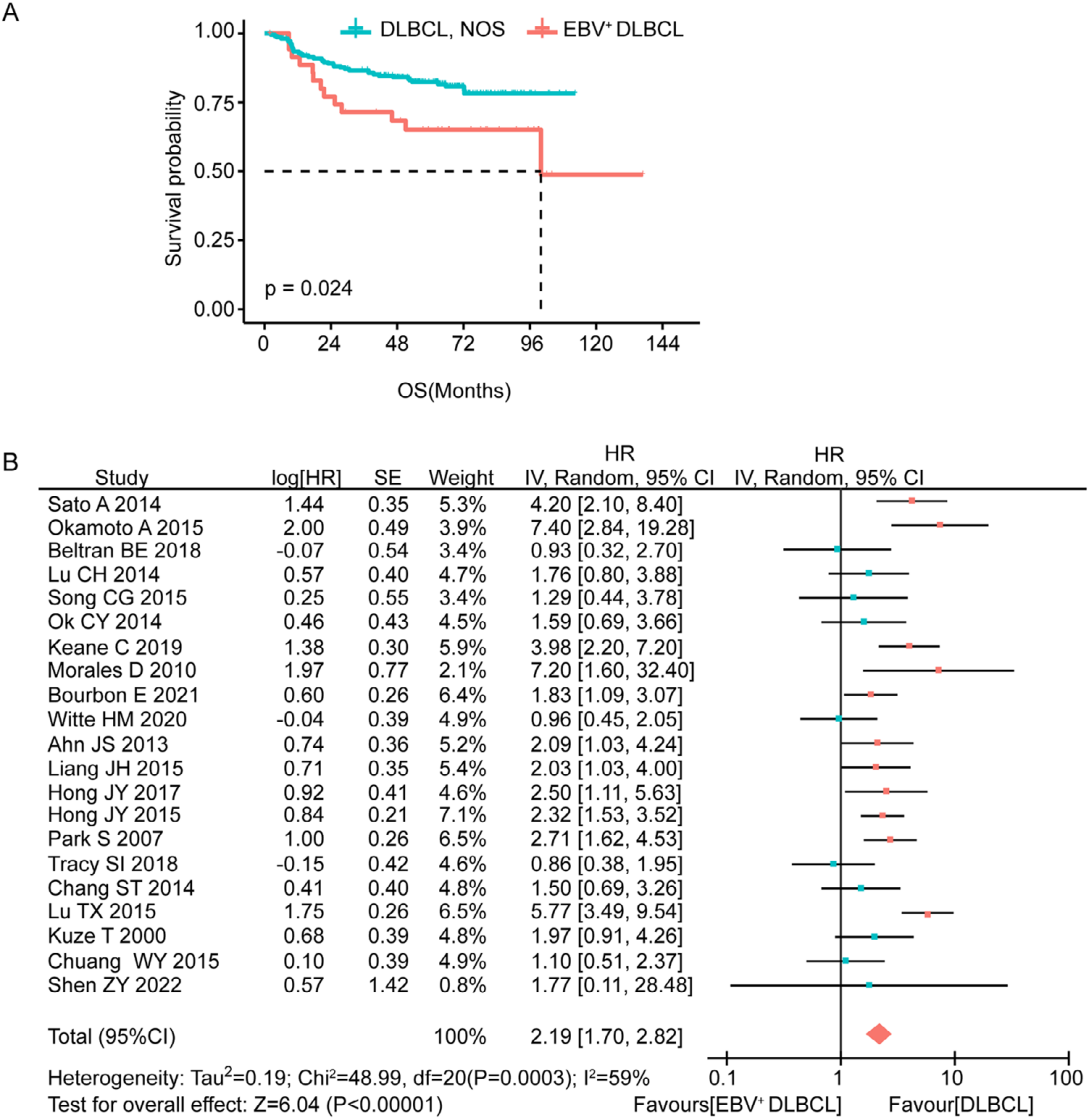
EBV^+^ DLBCL patients exhibited inferior overall survival compared to DLBCL, NOS patients. A) Kaplan–Meier survival curve comparing overall survival between 37 EBV^+^ DLBCL patients and 213 DLBCL patients, analyzed using the log‐rank test. B) Forest plot showing hazard ratios (HR) and 95% confidence intervals (CI) for overall survival (OS) in meta‐analysis. HR: Hazard Ratio. CI: Confidence Interval.

A total of 37 patients with EBV^+^ DLBCL and 213 patients with DLBCL, NOS were included in this study. The baseline characteristics of these patients are summarized in **Table** **1**. All patients received R‐CHOP‐like regimens (rituximab, cyclophosphamide, doxorubicin, vincristine, and prednisone) as first‐line chemotherapy. Response data were unavailable for seven EBV^+^ DLBCL patients. Among the evaluable cases, the overall response rate (ORR) after initial therapy was 92.5% (*n* = 197) in the DLBCL, NOS group and 78.4% (*n* = 29) in the EBV^+^ DLBCL group (*P* = 0.017, Table 1). The median OS time for patients with EBV^+^ DLBCL was 100 months, while the median OS for DLBCL, NOS patients was not reached. Besides, the 5‐year OS rate was significantly lower in the EBV^+^ DLBCL group than in the DLBCL, NOS group (65.1% vs 82.4%, *P* = 0.024) (**Figure** **1**A). To further compare the prognosis of EBV^+^ DLBCL and DLBCL, NOS, we performed a meta‐analysis. Details of the included studies and their Newcastle–Ottawa Scale scores are listed in Table S2 (Supporting Information). The pooled analysis showed that EBV^+^ DLBCL was associated with significantly worse overall survival (HR = 2.19, 95% CI: 1.70–2.82, *P*<0.00001) (Figure 1B). Moderate heterogeneity was observed across the studies (*I^2^* = 59%, *P* = 0.0003), so a random‐effects model was used to estimate the combined hazard ratio. These findings strongly suggest that EBV^+^ DLBCL is linked to a less favorable prognosis compared to DLBCL, NOS.

**Table 1:** Basine characteristics of DLBCL, NOS, and EBV^+^ DLBCL patients.

	DLBCL, NOS	EBV positive DLBCL	*P*‐value
	(*n* = 213)	(*n* = 37)	
Age (years)			
Median [IQR]	51.00 [40.00, 61.00]	50.00 [35.00, 58.00]	0.282
Sex			
Male	108 (50.7%)	23 (62.2%)	0.198
Female	105 (49.3%)	14 (37.8%)	
ECOG			
0‐1	195 (92.0%)	31 (96.9%)	0.532
2–3	17 (8.0%)	1 (3.1%)	
Molecular Subtype			
GCB	74 (35.9%)	1 (7.7%)	0.075
non‐GCB	132 (64.1%)	12 (92.3%)	
Stage			
I	32 (15.0%)	5 (13.5%)	0.453
II	84 (39.4%)	10 (27.0%)	
III	40 (18.8%)	9 (24.3%)	
IV	57 (26.8%)	13 (35.1%)	
IPI score			
0‐1	128 (60.1%)	16 (43.2%)	0.056
2–5	85 (39.9%)	21 (56.8%)	
LDH			
Elevated	86 (40.4%)	18 (50.0%)	0.279
Normal	127 (59.6%)	18 (50.0%)	
Extranodal involvement			
No	89 (41.8%)	15 (40.5%)	0.887
Yes	124 (58.2%)	22 (59.5%)	
B symptome			
No	179 (84.0%)	27 (73.0%)	0.103
Yes	34 (16.0%)	10 (27.0%)	
Bulky disease			
No	195 (91.5%)	33 (89.2%)	0.878
Yes	18 (8.5%)	4 (10.8%)	
Bone marrow involvement			
No	201 (95.3%)	28 (87.5%)	0.177
Yes	10 (4.7%)	4 (12.5%)	
Response to first line treatment			
ORR	197 (92.5%)	29 (78.4%)	0.017
DCR	204 (95.8%)	30 (81.1%)	0.003

Analyses were performed on available cases for each variable; for response evaluation, patients with “Unknown” status were retained in the denominator.

### 2.2 Establishment of Stable EBV Infection in Diffuse Large B‐Cell Lymphoma Cells

**Figure 2:**
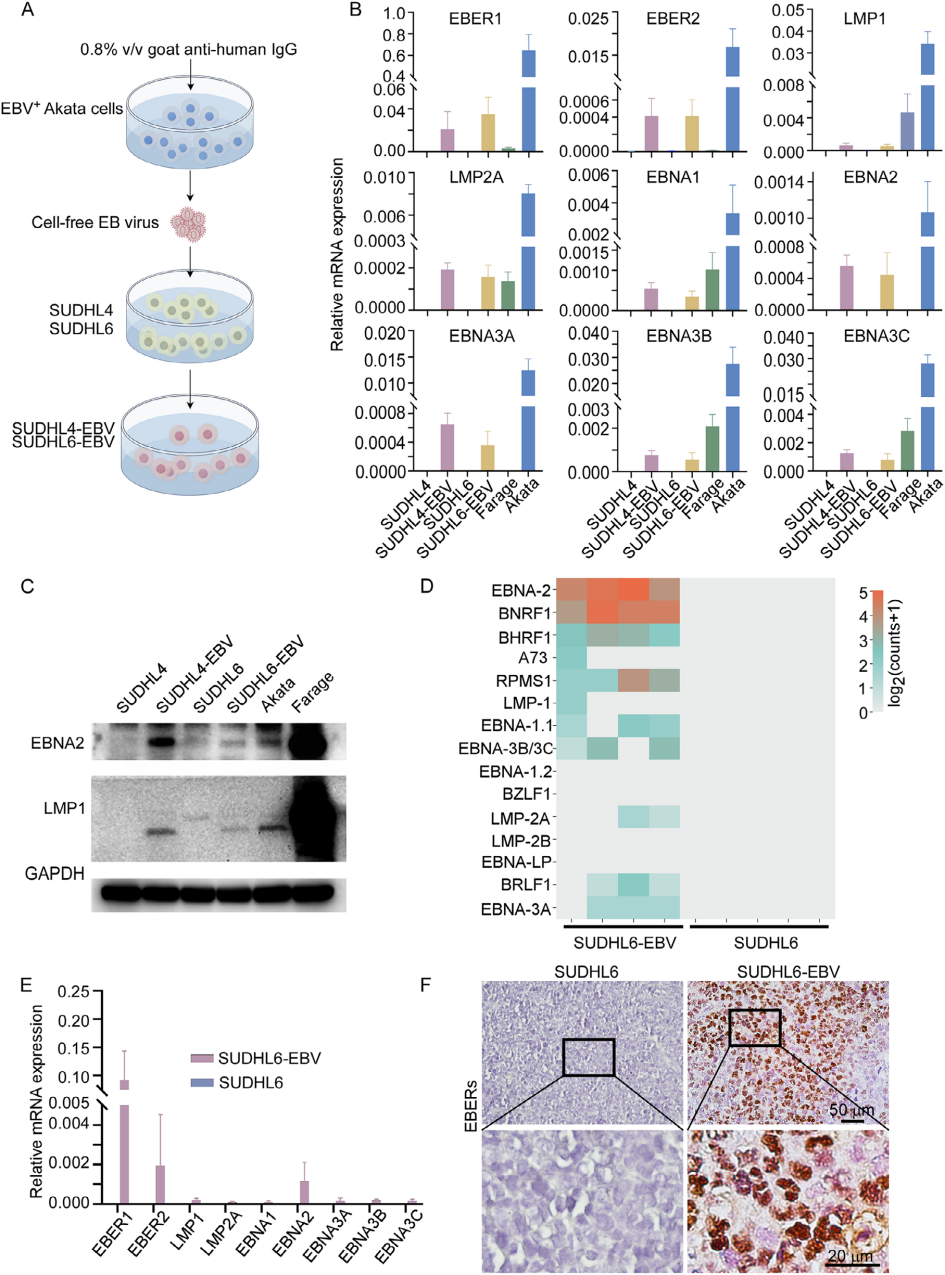
Establishment of Epstein‐Barr virus (EBV)‐infected diffuse large B‐cell lymphoma (DLBCL) cell lines. A) Schematic illustration showing the process of establishing EBV‐positive DLBCL cell lines. EBV‐negative DLBCL cell lines (SUDHL4 and SUDHL6) were infected with cell‐free EBV derived from Akata cells and subsequently selected using G418. This figure was created by Figdraw under copyright code‐[RURPI77371]. B) Quantification of latency gene expression in different cell lines by quantitative reverse transcription PCR (qRT‐PCR), including EBER1, EBER2, LMP1, LMP2A, EBNA1, EBNA2, EBNA3A, EBNA3B, and EBNA3C. Gene expression was normalized to β‐actin. C) Western blot analysis confirms the expression of EBNA2 and LMP1 in the indicated cell lines. Akata cells were stimulated with 0.8% goat anti‐human IgG for 6 h. D) Mapping analysis of transcript sequences from tumors derived from SUDHL6‐EBV or SUDHL6 cells against the Akata genome. E) Assessment of latency genes expression in xenograft tumors from EBV positive DLBCL cells and their parental counterparts. F) Representative images of EBERs‐ISH in tumor tissues from mice implanted with EBV‐positive DLBCL cells or their parental cells.

To better explore the biological behavior and characteristics of EBV^+^ DLBCL, we further established EBV^+^ DLBCL cell lines, as shown in **Figure** **2**A. After infection, EBV enters a latent state which can be categorized into four latency types‐latency III, II, I, and 0‐each characterized by distinct viral protein expression profiles.^[^ ^33^ ^]^ In EBV^+^ DLBCL patients, latency type II or III is commonly observed. Latency II is characterized by the expression of Epstein‐Barr nuclear antigen (EBNA)‐1, latent membrane protein (LMP)‐1, and LMP‐2, while latency III involves the expression of all latency genes. Remarkably, all nine latency genes were detected in the newly established EBV‐infected SUDHL4 (SUDHL4‐EBV) and SUDHL6 (SUDHL6‐EBV) cell lines (Figure 2B). Furthermore, both LMP1 and EBNA2 proteins were expressed in these infected cells (Figure 2C). Taken together, these results confirm that SUDHL4‐EBV and SUDHL6‐EBV cells exhibit latency III features at both the transcriptional level and the translational level.

To further validate the successful establishment of EBV‐infected cell lines, RNA sequencing was performed on tumors derived from both EBV^+^ and EBV‐negative SUDHL6 cells. Following the method described by Edwards RH's^[^ ^34^ ^]^, sequencing reads that could not be mapped to the human or mouse genomes were aligned to the Akata EBV genome to detect EBV‐specific transcripts. Multiple EBV genes were identified in the SUDHL6‐EBV group (Figure 2D), with EBNA2 and BNRF1 showing the highest expression levels. Moreover, the expression of EBV latency genes in xenografts was confirmed by RT‐qPCR. Both SUDHL6‐EBV and SUDHL4‐EBV xenografts exhibited expression of EBV latency genes (Figure 2E; Figure S1A, Supporting Information). Positive EBERs signals were also observed in SUDHL6‐EBV and SUDHL4‐EBV xenografts (Figure 2F; Figure S1B, Supporting Information). Collectively, these findings indicate the successful establishment of SUDHL4‐EBV and SUDHL6‐EBV cell lines.

### 2.3 EBV Infection Promotes the Proliferation of DLBCL In Vivo but not In Vitro

**Figure 3:**
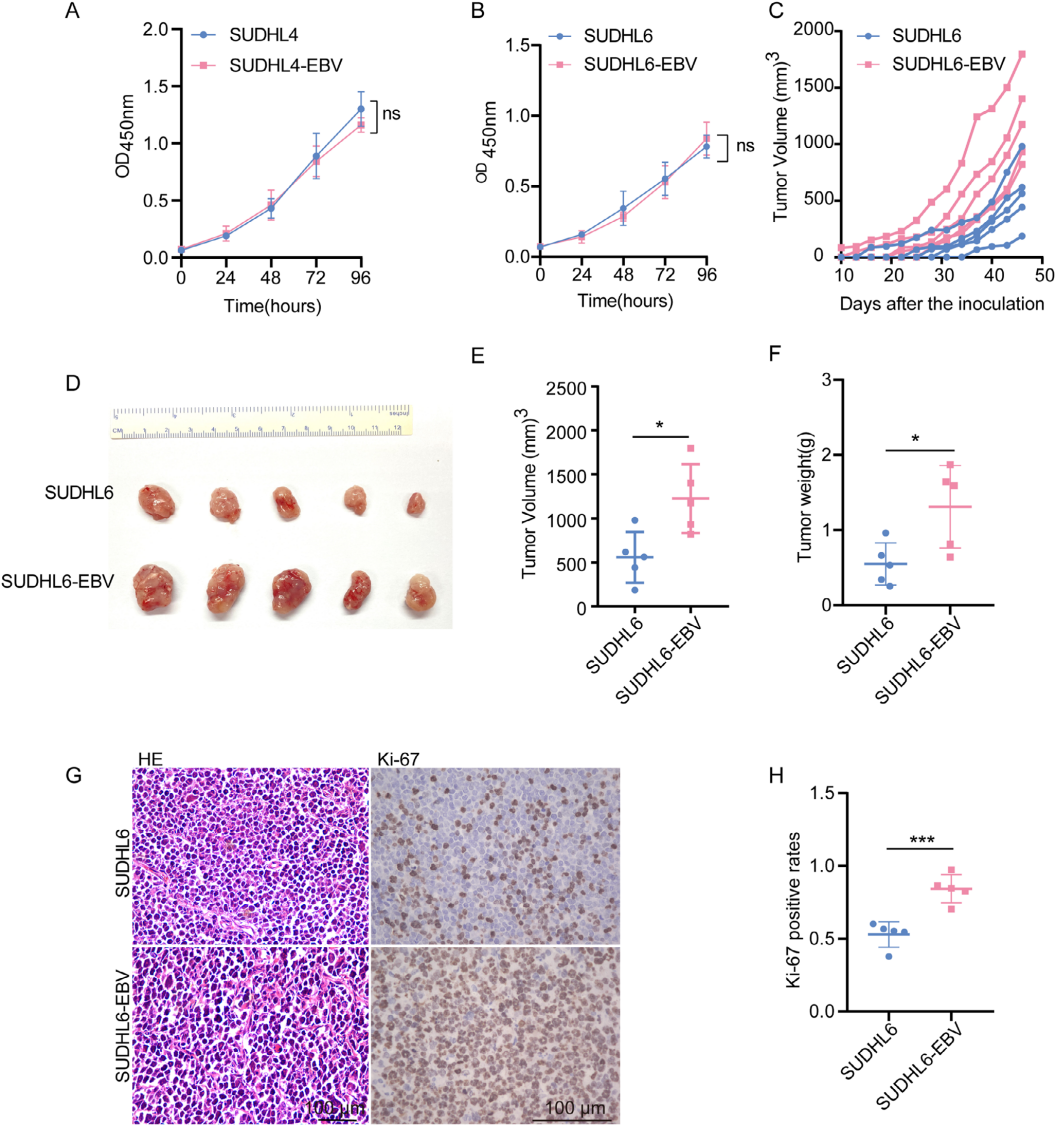
Comparing the biological behavior of EBV‐infected cells in vitro and in vivo. A, B) The proliferation of EBV‐infected cells and their parental cells was assessed in vitro using a cell counting kit‐8 assay. Data represent the results of three independent experiments and were analyzed using repeated measures ANOVA. *ns*: not significant. C–F) Equal numbers of SUDHL6 and SUDHL6‐EBV cells were subcutaneously injected near the inguinal lymph node of NOD/SCID mice. Tumor growth was monitored and compared, *n* = 5 mice per group. C) The tumor growth curve in NOD/SCID mice injected with either SUDHL6‐EBV cells or parental SUDHL6 cells. D–F) Representative images of tumor xenografts (D), tumor volumes (E), and tumor weights (F) of the tumor‐bearing mice on the day of sacrifice. Data were analyzed using Student's *t‐*test. ^*^ *P* < 0.05. G) Representative images of H&E and IHC staining for Ki‐67 in tumor tissues from each group. Scale bar, 100 µm. H) Quantification of Ki‐67 staining in the tumor tissues. Data were analyzed using Student's *t*‐test. ^***^ *P* < 0.001.

Previous studies have shown that EBV can transform B cells into lymphoblasts, leading to uncontrolled cell growth.^[^ ^35^ ^]^ However, its effect on the biological behavior of DLBCL cells still remains unclear. To this end, we initially compared the proliferation rates of EBV‐negative DLBCL cells and EBV‐infected DLBCL cells using CCK‐8 assays at 24, 48, 72, and 96 h after culture. Surprisingly, no significant difference in proliferation was observed between the two groups in vitro (**Figure** **3**A,B). Then, we investigated whether EBV infection affects tumor growth in vivo. EBV‐infected and parental DLBCL cells were subcutaneously injected near the inguinal lymph node of NOD/SCID mice. Strikingly, tumors derived from EBV‐infected DLBCL cells (SUDHL6‐EBV cells and SUDHL4‐EBV) grew significantly faster than those from their parental cells (Figure 3C–F; Figure S2A–D, Supporting Information). Furthermore, EBV‐infected DLBCL tumor tissues exhibited significantly higher Ki‐67 expression levels compared to those derived from parental cells (Figure 3G,H; Figure S2E,F, Supporting Information). In summary, our findings demonstrate that EBV promotes the proliferation of DLBCL in vivo but not in vitro.

### 2.4 EBV^+^ DLBCL Tumor Tissue has more Nerve Fiber Innervation

**Figure 4:**
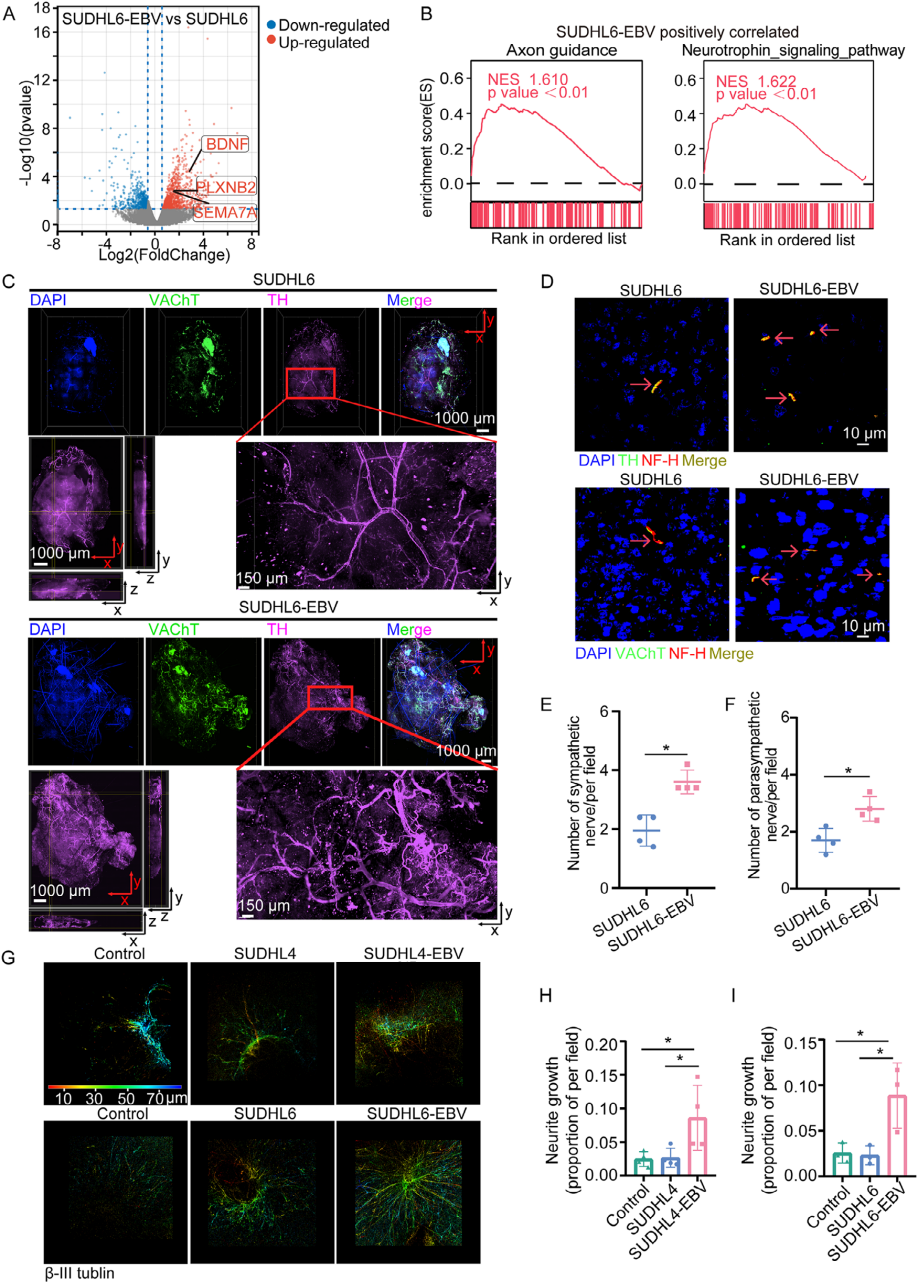
The infiltration of nerve fibers in DLBCL. A) Volcano plot showing gene expression differences between SUDHL6‐EBV and SUDHL6 xenograft tumor samples, based on fold change and *P* values. Blue and red dots represent significantly downregulated and upregulated genes, respectively. Genes related to axonogenesis are indicated. B) GSEA of xenograft tumors revealed significant enrichment of axon guidance and neurotrophin signaling pathways in SUDHL6‐EBV tumors. Enrichment scores (ES), normalized enrichment scores (NES), and *P* values (< 0.01) are shown. C) Representative 3D projection images of neural innervation in DLBCL xenograft tumors. Tumors were stained with VACHT (green), TH (purple), and DAPI (blue). D) Representative immunofluorescence (IF) images of nerve markers in murine tumor sections. Red arrows indicate nerve fibers. E, F) Quantification of nerve fiber density in tumor sections. *n* = 4 per group. Data were analyzed using the Mann–Whitney *u* test. ^*^ *P* < 0.05. G) Representative images of DRGs stained with β‐III tubulin after co‐culture with the indicated cell lines for 3 days. Color‐coded by penetration depth. H, I) Quantification of neurite growth, shown as a proportion of the field area, in DRG co‐cultures. Data are from at least three independent experiments and analyzed using One‐way ANOVA. ^*^ *P* < 0.05.

The observation that EBV infection promoted tumor growth in vivo not in vitro suggests that tumor microenvironment may play a key role in the progression of EBV^+^ DLBCL. To further identify microenvironmental factors that support tumor growth, we analyzed transcriptome sequencing data from tumor xenografts. Differential gene expression analysis showed that several genes related to axonogenesis were upregulated in EBV^+^ DLBCL xenografts (**Figure** **4**A). Additionally, GSEA revealed significant activation of the axon guidance and neurotrophin signaling pathways in EBV^+^ DLBCL (Figure 4B). To further explore this, we utilized whole‐tumor immunolabeling and optical clearing techniques to visualize the complete 3D morphology of nerve fibers in tumors formed by SUDHL6 and SUDHL6‐EBV cells in NOD/SCID mice. Tumor tissues were co‐stained with tyrosine hydroxylase (TH), a sympathetic‐neuronal marker, and vesicular acetylcholine transporter (VAChT), a parasympathetic‐neuronal marker. More nerve fiber innervation was observed in tumors derived from SUDHL6‐EBV cells (Figure 4C). To further confirm this phenomenon, we performed immunofluorescence staining on tumor sections using TH, VAChT, and neurofilament‐heavy (NF‐H) antibodies to quantify nerve fiber density. Consistently, tumors formed by EBV^+^ DLBCL cells showed abundant sympathetic and parasympathetic nerve fiber innervation (Figure 4D–F; Figure S3A, Supporting Information), suggesting that nerve fibers may contribute to the progression of EBV^+^ DLBCL.

To further figure out the role of EBV in nerve innervation, we established a co‐culture system using primary dorsal root ganglions (DRGs) and DLBCL cells (Figure S3B, Supporting Information). After 3 days of coculture, we evaluated axonal formation in DRGs using immunofluorescence staining with β‐III tubulin. Our results showed that DRGs co‐cultured with EBV‐infected DLBCL cell lines exhibited enhanced axonogenesis compared to controls and those cocultured with parental cells (Figure 4G–I). This observation was further corroborated in Farage cells, which are positive for EBV (Figure S3C,D, Supporting Information). Altogether, these findings indicate that EBV^+^ DLBCL tumor tissue has more nerve fiber innervation and possesses a greater capacity to attract nerve fibers into the tumor microenvironment.

### 2.5 Sympathetic Nerve Promotes the Proliferation of EBV^+^ DLBCL

**Figure 5:**
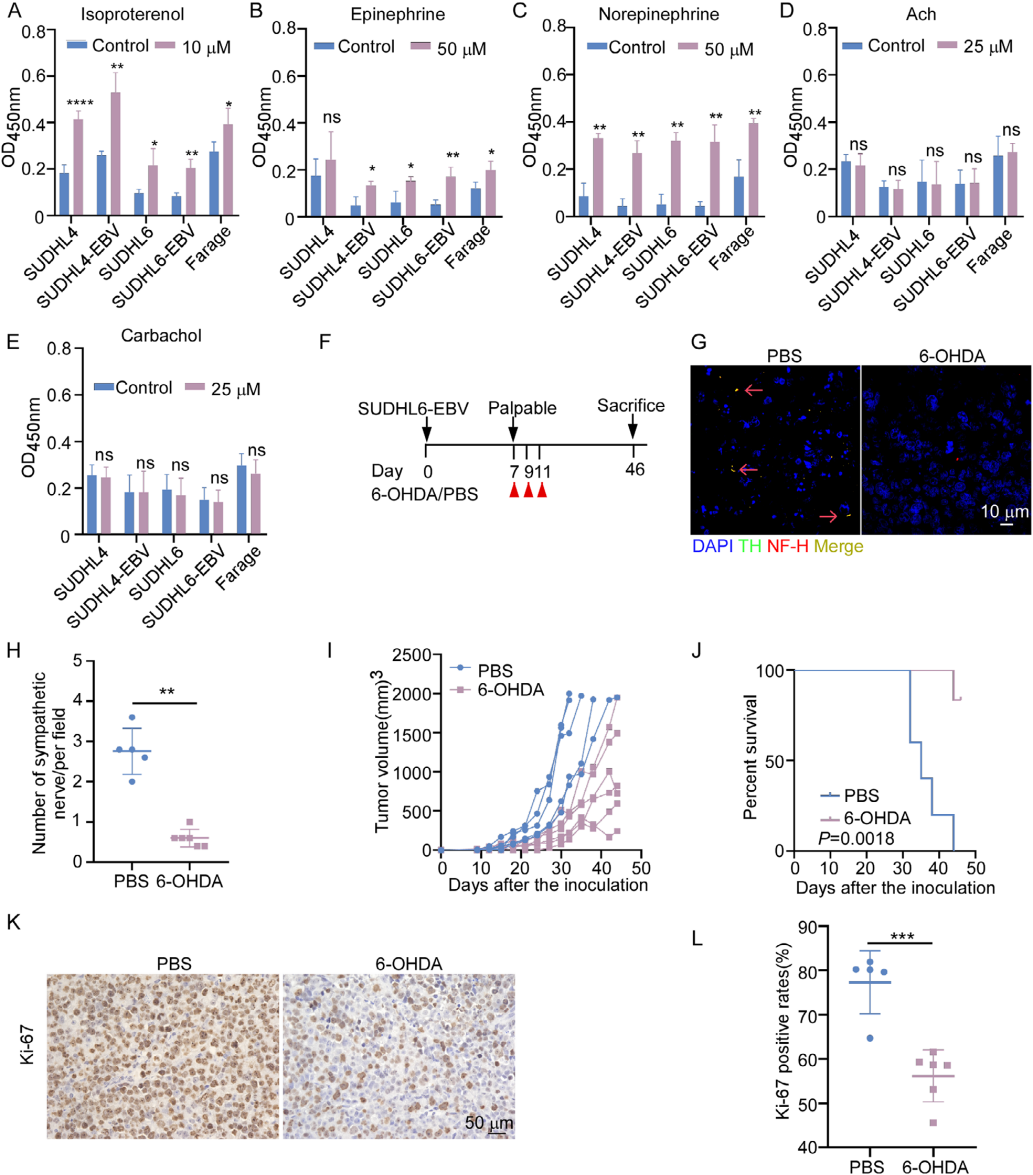
The sympathetic nerve promotes the proliferation of EBV^+^ DLBCL. A–C) The indicated cell lines were treated with three adrenergic neurotransmitters‐10 µm isoproterenol, 50 µm epinephrine, and 50 µm norepinephrine for 24 h. Cell proliferation was measured using the Cell Counting Kit‐8 assay (CCK‐8). Data are presented from at least three independent experiments and analyzed by Student's *t*‐test. ^*^ *P*<0.05, ^**^ *P*<0.01, ^****^ *P*<0.0001, *ns*: not significant. D, E) The same cell lines were treated with 25 µm cholinergic neurotransmitters, acetylcholine and carbachol, for 24 h. Cell proliferation was assessed using the CCK‐8 assay. Data are analyzed by Student's *t*‐test. *ns*: not significant. F–L) NOD/SCID mice bearing SUDHL6‐EBV tumors were treated with either PBS (*n* = 5) or 6‐OHDA (*n* = 6). Mice were sacrificed at the experimental endpoint. F) Experimental design showing 6‐OHDA‐induced peripheral sympathectomy. G, H) Representative immunofluorescent images (G) and quantification (H) of sympathetic nerve fibers in tumors from each group. Red arrows indicate nerve fibers. TH (green), NF‐H (red), DAPI (blue). Data were analyzed using the Mann–Whitney *u* test. ^**^P<0.01. Scale bar: 10 µm. I) Tumor growth curves of SUDHL6‐EBV tumors in the two groups. J) Survival curves of each group. Mice were monitored until death or when tumor volume approached 2000 mm^3^. Log‐rank test. K, L) Representative images (K) and quantification (L) of Ki‐67 staining in tumor tissues. Data were analyzed by Student's *t*‐test. ^***^ *P* < 0.001.

To investigate the possible effects of sympathetic and parasympathetic nerves on the progression of EBV^+^ DLBCL, we first treated both EBV^+^ and EBV‐negative DLBCL cells with three adrenergic neurotransmitters (isoproterenol, epinephrine, and norepinephrine) and two cholinergic neurotransmitters (acetylcholine and carbachol) in vitro. After 24 h, all three adrenergic neurotransmitters promoted tumor cell proliferation, while the cholinergic neurotransmitters had no significant effect (**Figure** **5**A–E). These findings suggest that adrenergic neurotransmitters may contribute to DLBCL cell growth.

To further confirm the role of sympathetic nerves in EBV^+^ DLBCL, we injected 6‐hydroxydopamine (6‐OHDA) into the tumor‐bearing mice to ablate adrenergic nerves (Figure 5F; Figure S4A, Supporting Information). Treatment with 6‐OHDA eliminated sympathetic nerve fibers (Figure 5G,H; Figure S4B,C, Supporting Information). Sympathectomy induced by 6‐OHDA led to a reduction in the growth of SUDHL6‐EBV tumors and an extension of overall survival (Figure 5I,J). Additionally, mice treated with 6‐OHDA had a lower Ki‐67 expression level in their xenografted tumors compared to mice treated with PBS (Figure 5K,L). Similarly, 6‐OHDA treatment also markedly suppressed the growth of SUDHL4‐EBV tumors (Figure S4D–G, Supporting Information). In contrast, blocking parasympathetic nerves using scopolamine (SCO), a non‐selective muscarinic receptor antagonist, had little effect on SUDHL6‐EBV tumor growth (Figure S4H,I, Supporting Information). Overall, these results highlight the important role of sympathetic nerves in promoting the growth of EBV^+^ DLBCL.

### 2.6 Sympathetic Nerve Promoted the Progression of EBV^+^ DLBCL via β2 Adrenergic Receptor

**Figure 6:**
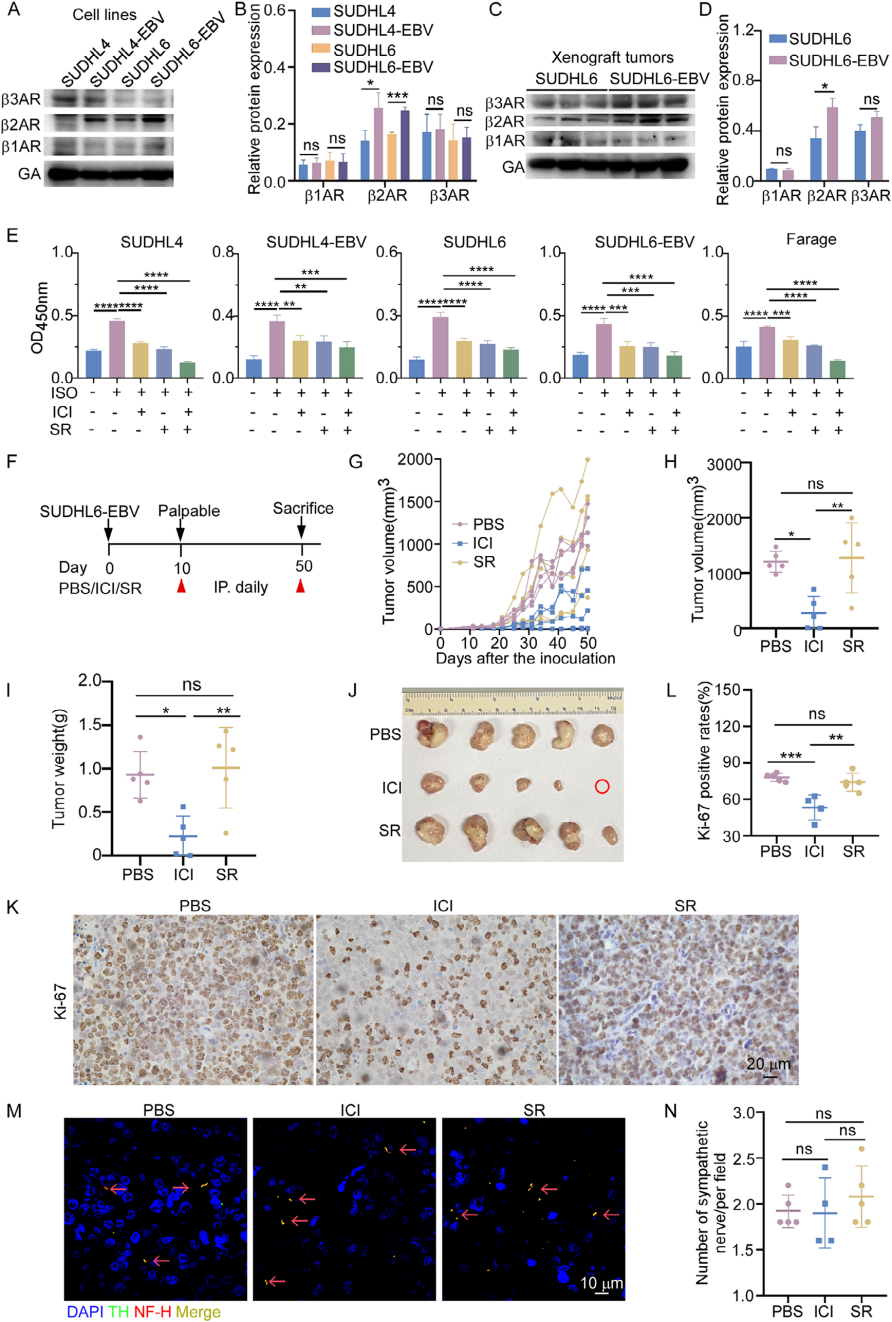
The sympathetic nerve promoted the growth of EBV^+^ DLBCL via β2 adrenergic receptor. A, B) Representative immunoblot images (A) and quantification (B) showing the expression of β‐adrenergic receptors (ADRBs) in the indicated cell lines. GA: GAPDH. Data are from three independent experiments and analyzed using Student's *t*‐test. ^*^ *P*<0.05, ^***^ *P* < 0.001, *ns*: not significant. C, D) Representative immunoblot images (C) and quantification (D) of ADRBs expression in tumor tissues from each group. *n* = 3 mice/group. Student's *t‐*test, ^*^ *P*<0.05, *ns*: not significant. E) The indicated cell lines were pre‐treated with 100 µm β2‐receptor blocker ICI‐118551 or 25 µm β3 receptor blocker SR59230A for 1 h, followed by treatment with 10 µm isoproterenol for 24 h. Cell proliferation was measured using the CCK‐8 assay. Data are from three independent experiments. One‐way ANOVA, ^**^ *P*<0.01, ^***^ *P *< 0.001, ^****^ *P*<0.0001. F–N) NOD/SCID mice bearing SUDHL6‐EBV were treated with PBS, ICI‐118551 (ICI), or SR59230A (SR), and sacrificed on day 50 post‐inoculation. *n* = 5 mice/group. F) Schematic diagram of the treatment protocol, showing daily injections of β2AR antagonist (ICI), β3AR antagonist (SR), or PBS as control. G) Tumor growth curves for each group. H, I) Tumor volume (H) and weight (I) on day 50. One tumor in the ICI‐treated group was not detectable. One‐way ANOVA, ^*^ *P*<0.05, ^**^ *P*<0.01. *ns*: not significant. J) Representative images of tumor xenografts on day 50. K, L) Representative images (K) and quantification (L) of Ki‐67 staining in tumor tissues. One sample in the ICI group was unavailable. Data were analyzed by one‐way ANOVA, ^**^ *P*<0.01, ^***^ *P* <0.001. M, N) Representative immunofluorescent images (M) and quantification (N) showing sympathetic nerve fibers in tumor tissues. Red arrows indicate nerve fibers. TH (green), NF‐H (red), DAPI (blue). One sample in the ICI group was unavailable. Data were analyzed using the Kruskal–Wallis test. *ns*: not significance. Scale bar: 10 µm.

Sympathetic nerves promote tumor growth through β‐adrenergic receptors (ADRBs), which are widely expressed in both normal and cancerous tissues.^[^ ^36^ ^]^ Our results indicated that DLBCL cells and tumors mainly express β2‐adrenergic receptors (β2ARs) and β3ARs, with β2AR levels increasing after EBV infection (**Figure** **6**A–D). Among these, β2AR is the main subtype found in human innate immune cells.^[^ ^37^, ^38^ ^]^ Sympathetic signaling has been reported to regulate lymphocyte dynamics and regulate adaptive immune responses through β2ARs.^[^ ^29^, ^30^ ^]^ To explore whether sympathetic neurotransmitters promote EBV^+^ DLBCL growth through β2ARs or β3ARs, we pre‐treated human DLBCL cell lines (SUDHL4 and SUDHL6), the EBV‐positive cell line Farage, and EBV‐infected cell lines (SUDHL4‐EBV and SUDHL6‐EBV) with selective antagonists: ICI‐118551 (β2AR blocker) and SR59230A (β3AR blocker). One hour later, the cells were treated with the βAR agonist isoproterenol. As expected, isoproterenol promoted DLBCL cell proliferation in vitro, but this effect was suppressed by pre‐treatment with either ICI‐118551 or SR59230A (Figure 6E).

To further identify the functional receptor in vivo, we inoculated EBV^+^ DLBCL cells into the inguinal lymph nodes of NOD/SCID mice. Once tumors became palpable, mice were randomly assigned to three groups and received daily intraperitoneal injections of either ICI‐118551 (10 mg kg^−1^), SR59230A (20 mg kg^−1^), or PBS as control (Figure 6F; Figure S5A, Supporting Information). Notably, the selective β2AR antagonist ICI‐118551, but not the β3AR antagonist SR59230A, inhibited EBV^+^ DLBCL tumor growth (Figure 6G–L, Figure S5B–E, Supporting Information). Furthermore, neither ICI‐118551 nor SR59230A altered the density of TH⁺ nerve fibers within the tumor microenvironment, thereby excluding the possibility of a reduction in sympathetic nerve fibers (Figure 6M,N; Figure S5F,G, Supporting Information). In summary, these results demonstrate that the sympathetic nerves promote EBV^+^ DLBCL progression through β2AR signaling and that blocking β2ARs with a selective antagonist can effectively suppress tumor growth.

### 2.7 More Sympathetic Nerve Infiltrated in EBV^+^ DLBCL and Correlated with Inferior Survival

**Figure 7:**
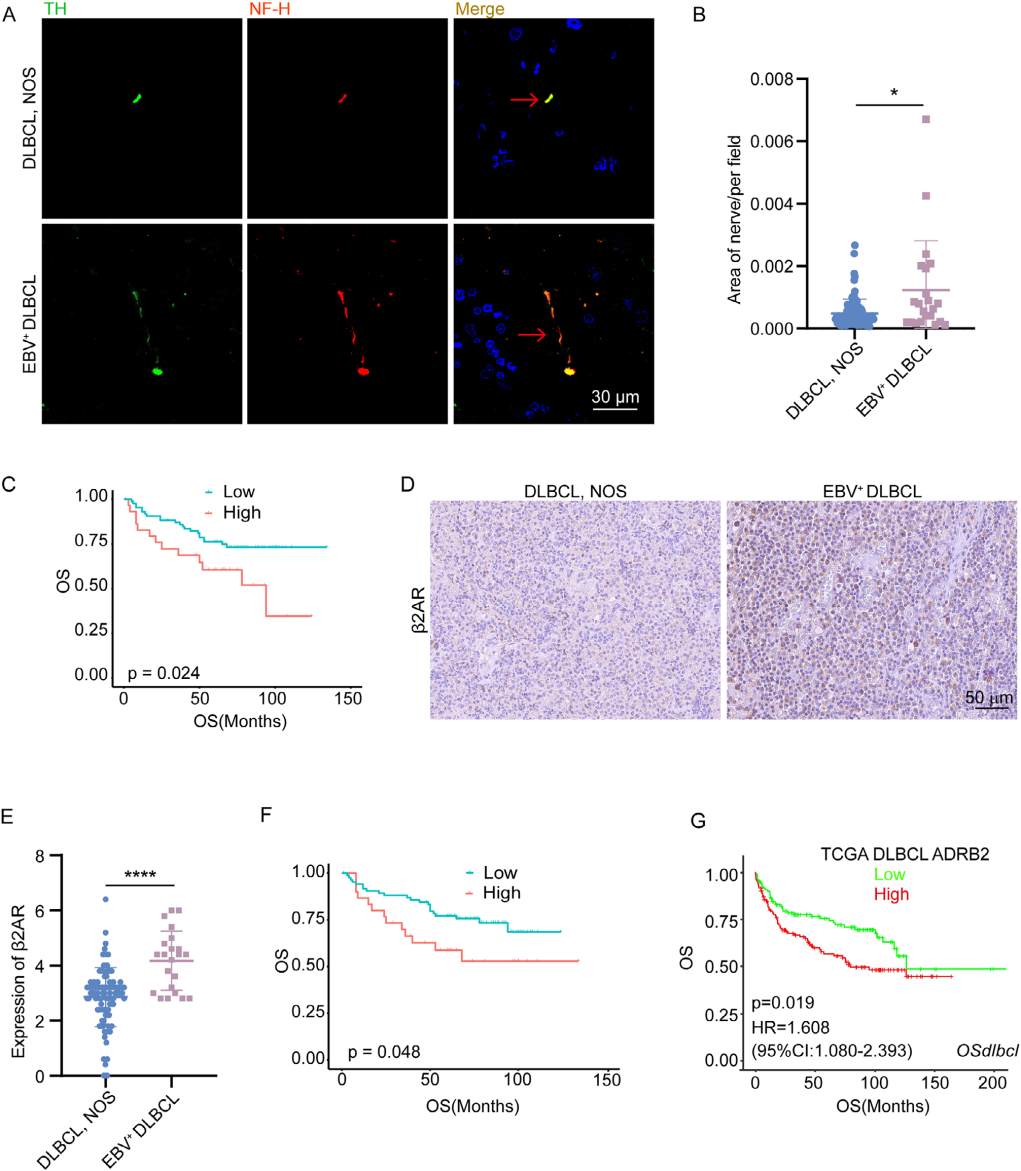
Increased sympathetic nerve fiber infiltration in EBV⁺ DLBCL is associated with poorer survival. A) Representative immunostaining images showing sympathetic nerve fibers in the tumor tissues from patients with DLBCL, NOS (*n* = 93), and EBV^+^ DLBCL (*n* = 22). Red arrows indicate nerve fibers. B) Quantification of sympathetic nerve in the tumor microenvironment based on immunostaining of patient samples. Data were analyzed using Student's *t*‐test with Welch's correction, ^*^ *P*<0.05. C) Kaplan–Meier overall survival curve for patients with a low group (*n* = 86) and high TH expression (*n* = 29). *P* value was calculated using the log‐rank test. D) Representative immunostaining images showing β2AR expression in tumor tissues of DLBCL, NOS (*n* = 93) and EBV^+^ DLBCL patients (*n* = 22). Scale bar: 50 µm. E) Quantification of β2AR using the Allred scoring method. ^****^ *P*<0.0001, Mann–Whitney *u* test. F) Kaplan–Meier survival analysis comparing overall survival in patients with low (*n* = 85) and high (*n* = 30) β2AR expression. *P* value was calculated using the log‐rank test. G) Kaplan–Meier survival curve based on ADRB2 mRNA expression levels in DLBCL patients from the TCGA database. Survival analysis was performed using the OSdlbcl online tool, and the *P* value was determined by the log‐rank test.

These findings prompted us to further investigate the clinical relevance of sympathetic nerve innervation and β2AR expression. We performed immunofluorescence (IF) staining on tumor tissues from 93 patients with DLBCL, NOS, and 22 patients with EBV^+^ DLBCL (**Figure** **7**A). EBV^+^ DLBCL patients exhibited significantly higher levels of sympathetic nerve infiltration compared to those with DLBCL, NOS (Figure 7B). Moreover, a high level of TH was associated with remarkably worse OS (Figure 7C). Besides, elevated β2AR levels were also observed in EBV^+^ DLBCL tissues (Figure 7D,E). Patients with high β2AR expression exhibited poorer OS than those with low β2AR expression (Figure 7F). Notably, in The Cancer Genome Atlas (TCGA) database, high mRNA expression levels of ADRB2 (which encodes β2AR) were significantly correlated with shorter OS in DLBCL patients, as shown in Figure 7G, generated using the OSDLBCL online analysis platform.^[^ ^39^ ^]^ Taken together, these findings demonstrate that EBV^+^ DLBCL tumors exhibit increased infiltration of sympathetic nerves, which is associated with poor survival.

Although sympathetic nerves are known to promote the progression of various cancers, including breast, prostate, and pancreatic cancers via β‐adrenergic receptors^[^ ^15^, ^17^, ^40^, ^41^ ^]^, and β‐adrenergic receptor (βAR) blockers are widely used in clinical settings, their impact on cancer survival remains controversial. Therefore, we conducted a meta‐analysis to evaluate the correlation between βAR blockers and cancer prognosis. Details of the included studies and their Newcastle–Ottawa Scale scores are provided in Table S3 (Supporting Information). This meta‐analysis incorporated 658,528 participants: 373,022 from breast cancer studies, 107,194 from prostate cancer studies, 156,981 from colorectal cancer studies, and 21,331 from pancreatic cancer studies. The pooled results showed a significant reduction in the risk of cancer‐specific mortality among individuals who use beta‐blockers compared to non‐users (HR = 0.94, 95% CI: 0.90‐0.99; *P* = 0.02; Figure S6, Supporting Information). Thus, these results highlight the potential therapeutic benefits of targeting β‐adrenergic receptors in cancer treatment.

## 3 Discussion

EBV^+^ DLBCL is a rare and aggressive subtype characterized by consistently poor outcomes in the R‐CHOP era, as confirmed by our study and previous reports.^[^ ^42^, ^43^, ^44^, ^45^ ^]^ Using our newly established EBV^+^ DLBCL cell lines, we demonstrated that EBV infection accelerated in vivo tumor growth without affecting in vitro proliferation. Notably, we observed increased sympathetic nerve innervation in tumors derived from SUDHL6‐EBV cells, and β2AR antagonists effectively inhibited tumor progression. Importantly, we provided the first clinical evidence that EBV^+^ DLBCL patients showed elevated sympathetic nerve density and β2AR expression in tumor tissues, both of which were significantly correlated with worse prognosis. Furthermore, the use of beta‐blockers was linked to reduced cancer‐specific mortality, thereby supporting the therapeutic potential of β2AR antagonists for EBV^+^ DLBCL.

Robust research models are essential for exploring the underlying mechanisms of EBV^+^ DLBCL and for developing new strategies to improve patient outcomes. Establishing EBV^+^ DLBCL cell lines offers valuable tools for gaining deeper insights into disease pathogenesis. Consistent with Stefan's study^[^ ^46^ ^]^, we observed no difference in cell proliferation between EBV‐positive and EBV‐negative DLBCL cells in vitro. However, in vivo, EBV‐infected DLBCL cells exhibited markedly faster tumor growth compared to their parental counterparts, despite similar in vitro behavior. These findings highlight the distinct biological behavior of EBV^+^ DLBCL in vivo and emphasize the critical role of the tumor microenvironment in disease progression.

Our study revealed increased sympathetic nerve infiltration in EBV^+^ DLBCL tumors. Transcriptome analysis showed upregulation of *brain‐derived neurotrophic factor (BDNF)*, *semaphorin 7A (SEMA7A)*, and *plexin‐B2 (PLXNB2)* in SUDHL6‐EBV derived tumor tissues. BDNF, a well‐characterized neurotrophin, stimulates axonal elongation and branching through activation of its receptor TrkB and downstream PI3K/Akt and MAPK signaling pathways.^[^ ^47^, ^48^ ^]^ Similarly, SEMA7A, originally identified as an immune modulator, promotes neurite outgrowth by binding to integrins and activating MAPK signaling.^[^ ^49^ ^]^ PLXNB2 serves as a receptor for semaphorins.^[^ ^50^, ^51^ ^]^ Their upregulation suggests that EBV^+^ DLBCL tumors may promote sympathetic nerve innervation by releasing axon guidance factors. Future investigations are warranted to determine whether these specific molecules drive sympathetic innervation in EBV^+^ DLBCL. In vitro experiments revealed that blockade of both β2AR and β3AR inhibited tumor growth, whereas in vivo studies demonstrated that only β2AR antagonism effectively suppressed tumor progression. This discrepancy may reflect reduced β3AR expression levels in the tumor microenvironment or compensatory mechanisms present in vivo. While the impact of β‐blockers on cancer survival remains controversial^[^ ^52^, ^53^, ^54^, ^55^ ^]^, our meta‐analysis identified a modest yet statistically significant survival benefit among patients treated with β‐blockers. Notably, β2AR signaling has been implicated in the progression of multiple cancers^[^ ^15^, ^17^, ^40^, ^56^, ^57^, ^58^, ^59^ ^]^, and our work now extends this paradigm to EBV^+^ DLBCL‐representing the first evidence of nerve‐driven tumor progression in lymphoma. These findings highlight β2AR as a therapeutic target and underscore the need to dissect nerve‐tumor crosstalk in lymphoid malignancies.

Despite our efforts, this study has several limitations. First, the sample size of EBV^+^ DLBCL was relatively small, and larger studies are needed to confirm our findings. Additionally, although we observed increased sympathetic nerve infiltration and its potential role in promoting EBV^+^ DLBCL progression, further research is necessary to elucidate the underlying mechanisms.

## 4 Conclusion

Our study reveals sympathetic nerve infiltration as a novel pathological feature of EBV^+^ DLBCL, demonstrating that β2AR signaling promotes tumor progression in this aggressive lymphoma subtype. These findings establish β2AR as a promising therapeutic target for EBV^+^ DLBCL, addressing the critical need for more effective treatments. Furthermore, the identification of nerve‐tumor crosstalk in lymphoma paves the way for novel mechanistic studies and targeted therapies.

## 5 Experimental Section

### Patient Samples and Data Collection

768 patients diagnosed with de novo DLBCL at Sun Yat‐Sen University Cancer Center were retrospectively reviewed to identify those who had undergone Epstein‐Barr virus‐encoded small RNA (EBER) in situ hybridization (ISH) testing. A total of 250 patients with available EBER‐ISH results were included for overall survival (OS) analysis, among whom 37 were diagnosed with EBV^+^ DLBCL. The paraffin‐embedded tissue samples were collected for further analysis (DLBCL, NOS: *n* = 93; EBV^+^ DLBCL: *n* = 22). The study was approved by the Institutional Review Board of Sun Yat‐sen University Cancer Center (Approval numbers: B2022‐141) and was conducted in accordance with the Declaration of Helsinki, relevant laws, and institutional guidelines. Informed consent was obtained from all participants.

To evaluate baseline clinical characteristics, clinicopathological data were reviewed, including age, sex, Eastern Cooperative Oncology Group (ECOG) performance status, disease stage, International Prognostic Index (IPI) score, lactate dehydrogenase (LDH) level, presence of B symptoms, extranodal involvement, molecular subtype, presence of bulky disease, bone marrow involvement, and response to first‐line treatment.

### Systematic Review of Prognosis in EBV^+^ DLBCL versus DLBCL and the Impact of Beta‐Blocker Use on Cancer Outcomes

A comprehensive literature search across several electronic databases, including PubMed, Embase, the Cochrane Library, and Web of Science, covering studies published before August 15, 2024 was conducted. Details of the search strategy and selection criteria are provided in the Section S1 (Supporting Information). Two independent reviewers extracted data based on predefined criteria, including study design, patient characteristics, and survival outcomes. The quality of each included study was independently assessed by two reviewers using the Newcastle–Ottawa Scale. Studies with Newcastle–Ottawa Scale scores greater than six were considered to be of moderate to high quality. OS and cancer‐specific mortality were selected as the primary endpoints for this meta‐analysis. Hazard ratios (HRs) and 95% confidence intervals (CIs) were either directly obtained from the original articles or indirectly estimated from Kaplan–Meier curves using software developed by Tierney et al.

### Cell Lines

The human diffuse large B‐cell lymphoma cell lines SUDHL4, SUDHL6, and Farage were obtained from the American Type Culture Collection (ATCC, Manassas, VA, USA). The human Burkitt lymphoma cell line Akata, which carried recombinant EBV, was kindly provided by Prof. Musheng Zeng at Sun Yat‐sen University Cancer Center (China). SUDHL4, SUDHL6, and Farage were cultured in RPMI 1640 medium (Gibco, Carlsbad, CA) supplemented with 10% fetal bovine serum (FBS), while Akata cells were maintained in RPMI 1640 medium containing 5% FBS. All cell lines were incubated at 37 °C in a humidified atmosphere with 5% CO₂. The identity of the cell lines was confirmed by short tandem repeat (STR) profiling.

### Virus Production

Virus production was performed according to previously described methods.^[^ ^60^ ^]^ Briefly, EBV‐positive Akata cells were suspended in FBS‐free RPMI 1640 medium at a density of 2 × 10^6^ cells mL^−1^ and induced with 0.8% (v/v) of goat anti‐human immunoglobulin G serum (Cat#H0111‐6, Shuangliu Zhenglong Biochem.Lab) for 6 h at 37 °C. After induction, the cells were transferred to RPMI 1640 containing 3.5% FBS and incubated for 3 days. The supernatant was then collected, centrifuged (4000 rpm, 10 min), filtered (0.8 and 0.45 µm), and ultracentrifuged (25,000 rpm, 3 h, 4 °C). The resulting virus was resuspended in serum‐free RPMI 1640 and stored at −80 °C until use.

### Cell‐Free EBV Infection

SUDHL4 and SUDHL6 cells were harvested and resuspended in FBS‐free RPMI 1640 medium. Next, 5 × 10⁴ cells were seeded into each well of a 96‐well plate. Then, 200 µL of recombinant EBV‐containing supernatant was gently added to infect EBV‐negative SUDHL4 and SUDHL6 cells. The plates were centrifuged at 1000 rpm for 1.5 h and incubated at 37 °C for 24 h. On the following day, the viral supernatant was removed, and the cells were cultured in a fresh medium for another 48 h. To select successfully infected cells with neomycin resistance, a G418‐containing medium (8000 µg mL^−1^ for SUDHL4 and 2000 µg mL^−1^ for SUDHL6) was added. Infected cells expressing GFP were identified under a fluorescence microscope (Olympus).

### Quantitative Reverse Transcription Polymerase Chain Reaction

Quantitative reverse transcription polymerase chain reaction (qRT‐PCR) was performed according to previously described protocols.^[^ ^61^ ^]^ Briefly, total RNA was extracted from tumor samples or cell lines using TRIzol reagent (Cat#15596018CN, Invitrogen). Then, 500 ng of total RNA was reverse transcribed into cDNA using PrimeScript RT Master Mix (Cat#RR036A, TAKARA), following the manufacturer's instructions. PCR amplification was carried out in 96‐well plates using SuperReal Premix Plus (SYBR Green, Cat#RR820A, TAKARA) on a CFX96 Real‐Time PCR System (Bio‐Rad). Primers were obtained from Generay. All reactions were performed in triplicate. The relative mRNA expression of the target genes was normalized to β‐actin. The primer sequences are listed in Table S1 (Supporting Information).

### Western Blot

Cells were placed on ice for 30 min in 100 µL of RIPA buffer containing protease inhibitor (Cat#CW2200S, CWBIO) and phosphatase inhibitor (Cat#CW2383S, CWBIO). After lysis, cell debris was removed by centrifugation at 1 4000 rpm for 15 min. Protein concentrations were measured using the BCA protein assay kit (Cat#23225, Thermo) and normalized to equal protein amounts. Protein samples were separated by 10% SDS‐PAGE and transferred to PVDF membranes. The membranes were incubated with primary antibodies against EBNA2 (Cat#ab90543; Abcam), LMP1 (Cat#ab78113, Abcam), and GAPDH (Cat#5174, CST), ADRB1 (Cat#MAB10119, R&D), ADRB2 (Cat#ab182136, Abcam) and ADRB3 (Cat#sc‐518080, Santa Cruz). Species‐specific HRP‐conjugated secondary antibodies (Cell Signaling) were then applied. The immunoreactive bands were visualized using an enhanced chemiluminescence kit (Cat#180‐5001W, Tanon). Band intensities were quantified using ImageJ software and normalized to the corresponding GAPDH band.

### Histology and Immunohistochemistry

Tissue samples were fixed in 10% formalin, dehydrated, embedded in paraffin, sectioned, and stained with hematoxylin and eosin. For immunohistochemistry (IHC), 5 µm‐thickness paraffin sections were prepared. The sections were dewaxed using an eco‐friendly dewaxing agent (Cat#H‐H0102, Huntz) and rehydrated with ethanol. Heat‐induced antigen retrieval was performed by microwave heating at high power for 5 min, followed by medium‐low power for 20 min in 0.01 m ethylenediaminetetraacetic acid buffer (pH 9.0). Subsequently, endogenous peroxidase activity was blocked using 3% hydrogen peroxide for 10 min. Nonspecific binding was blocked with 5% BSA for 30 min. Sections were incubated overnight at 4 °C with primary antibodies against Ki‐67 (Cat#ab16667, Abcam), ADRB2 (Cat#ab182136, Abcam). Immunostaining was carried out using a DAB detection system (Cat#GK500710, Gene tech) according to the manufacturer's instructions. The intensity of Ki‐67 staining was quantified using ImageJ. The expression levels of ADRB2 were scored based on staining intensity and positive rate using the Allred scoring method as previously described.^[^ ^62^ ^]^ Briefly, Allred score = SI (staining intensity) + PP (percentage of positive cells). SI was assigned as: 0 = negative; 1 = weak; 2 = moderate; and 3 = strong. PP is defined as 0 = 0%; 1 = 0%–25%; 2 = 25%–50%; 3 = 50%–75%; 4 = 75%–100%.

### Immunofluorescence

Paraffin‐embedded tumor sections were deparaffinized and subjected to antigen retrieval using ethylenediaminetetraacetic acid (EDTA) buffer (pH 9.0), as described above. The sections were then blocked with 5% BSA for 30 min at room temperature. Next, the sections were incubated overnight at 4 °C with primary antibodies, including VAChT (Cat#SAB4200559‐200UL, Sigma), TH (Cat#PA1‐4679, Thermo), NF‐H (Cat#60331‐1‐Ig, Proteintech). After washing with PBS, the sections were incubated for 60 min at room temperature with the following secondary antibodies, as appropriate: Alexa Fluor 555‐conjugated anti‐mouse IgG (Cat#A32773, Thermo), Alexa Fluor 488‐conjugated anti‐rabbit IgG (Cat#ab150177, Abcam) or Alexa Fluor 647‐conjugated anti‐sheep IgG (Cat#ab150075, Abcam). Following three times of PBS washing, the sections were incubated with DAPI (Cat#C1002, Beyotime) for 30 min at room temperature. After washing with PBS, the sections were mounted using a Vectashield mounting medium. Nerve density in the tumor tissue was presented as the average number from five randomly selected fields at ×63 magnification.

### EBERs In Situ Hybridization

EBERs were detected using an in situ hybridization kit according to the manufacturer's instructions (Cat#ISH‐7001, ZSGB‐Bio). Initially, 5 µm paraffin‐embedded sections were baked at 60 °C for 3 h, then deparaffinization using standard protocols. The sections were then hybridized with EBV‐specific oligonucleotide probes targeting EBERs. The hybridization signals were visualized using a DAB detection system.

### Cell Proliferation Assay

For the cell proliferation assays, the indicated cell lines were seeded into 96‐well plates at a density of (5‐10) ×10^3^ per well in growth medium and cultured for various time intervals. To examine the effect of neurotransmitters, cells were seeded in 96‐well plates at the same density and treated for 24 h with the following compounds: 10 µm isoproterenol sulfate (ISO, β‐AR agonist, Cat#S5683, Selleck), 50 µm epinephrine bitartrate (α/β‐adrenergic receptors agonist, Cat#S2521, Selleck), 50 µm norepinephrine (α/β‐adrenergic receptors agonist, Cat#S9507, Selleck), 25 µm Ach (Cat#S1805, Selleck) and 25 µm Carbachol (Cat#S4359, Selleck) (Acetyl‐cholinergic receptor agonists). To assess the effect of β‐AR antagonist, cells were seeded as described above and pretreated for 2 h with 100 µm ICI‐118551 hydrochloride (ICI, β2‐AR selective antagonist, Cat#S8114, Selleck) and 25 µm SR59230A (β3‐AR selective antagonist, Cat#S0812, Selleck) followed by treatment with 10 µm ISO for 24 h. Cell proliferation was measured using the Cell Counting Kit‐8 assay (CCK8, Cat#cck8‐100‐120, GOONIE) according to the manufacturer's instructions. Absorbance was read at 450 nm using a microplate reader (Bio‐Tek). Values were adjusted by subtracting background readings (blank control), and each condition was tested in triplicate.

### Preparation of Dorsal Root Ganglions

Dorsal root ganglions (DRGs) were harvested from 3‐week‐old Sprague Dawley (SD) rats as previously described. Briefly, the rats were euthanized and sterilized with alcohol. After removing the skin and soft tissue in the back, the spine was carefully exposed to reveal the spinal nerves and DRGs. The DRGs were then dissected bilaterally and transferred into a 96‐well plate containing DMEM/F12 (Gibco). The anterior and posterior nerve roots attached to the DRG were carefully trimmed.

### Neurite Outgrowth Assay of DRG In Vitro

DRGs, embedded in growth factor‐depleted Matrigel, were implanted at the bottom of the 6‐well plate, where a coverslip had been placed in advance. The indicated cell lines were suspended in 30 µL Matrigel (Cat#354234, Corning) at a density of 2 × 10^5^ cells and carefully placed around the DRG. The coculture system was maintained in RPMI 1640 medium supplemented with 10% fetal bovine serum at 37 °C in a 5% CO_2_ incubator for 3 days. After coculturing, the coverslips were subjected to immunofluorescence staining using β‐III tubulin and imaged.

### Immunofluorescence of DRG

This procedure was performed as previously described.^[^ ^23^ ^]^ Briefly, Matrigel‐embedded DRGs were fixed in 4% paraformaldehyde overnight at 4 °C, then blocked with 10% horse serum and 1% Triton X‐100 in PBS for 4 h at room temperature. After blocking, the DRGs were incubated overnight at 4 °C with the primary antibody against β‐III tubulin (Cat#5568, CST) diluted in 5% horse serum with 0.01% Triton X‐100. The samples were then washed with PBS and incubated with Alexa Fluor 488‐conjugated anti‐rabbit IgG for 90 min at room temperature. After another PBS wash, the samples were mounted with a Vectashield mounting medium. Confocal images were acquired using a laser‐scanning microscope (LSM880, Carl Zeiss). Neurite outgrowth from DRGs was quantified using ImageJ by calculating the total neurite area, expressed as a proportion of the total image area.

### Animal Models

NOD/SCID mice were purchased from Beijing Vital River Laboratory Animal Technology and housed in a specific pathogen‐free facility. To compare tumor growth between EBV‐negative and EBV‐infected DLBCL cells, equal numbers of lymphoma cells were subcutaneously injected into the flank of each 6–8‐week‐old NOD/SCID mouse, near the inguinal lymph node. Specifically, 100 µL of serum‐free medium (comprising 50 µL PBS and 50 µL matrigel), containing 5×10^6^ DLBCL cells, was injected subcutaneously near the inguinal lymph node of each mouse.

6‐OHDA is a selective neurotoxin for sympathetic nerves that can effectively ablate sympathetic nerves without affecting VAChT^+^ parasympathetic fibers. It was widely used in cancer neuroscience studies.^[^ ^63^, ^64^ ^]^ For sympathetic nerve denervation of tumors, once the tumor became palpable after the injection of EBV‐infected DLBCL cells, the mice were randomly assigned to two groups and received either 6‐OHDA (Cat#H4381, Sigma–Aldrich) or PBS, as previously described.^[^ ^17^ ^]^ 6‐OHDA or PBS was administered on the first day (100 mg kg^−1^), third day (200 mg kg^−1^), and fifth day (250 mg kg^−1^).

For experiments involving adrenergic receptor blockers, once the tumor became palpable after the injection of EBV‐infected DLBCL cells, animals received either PBS alone, the selective β2‐adrenergic receptor blocker ICI‐118551 at 10 mg kg^−1^ per day alone, or the selective β3‐adrenergic receptor blocker SR59230A at 20 mg kg^−1^ per day alone.

Tumor sizes were monitored by measuring their length and width every 3 days, and volume was calculated using the formula: (length) × (width)^2^ × 0.5. Data are presented as means ± standard deviation. Mice were euthanized when tumors approached a maximum volume of 2000 mm^3^, and tumors were harvested for further analysis (The maximal tumor size permitted by the ethics committee was 2000 mm^3^).

All animal procedures were conducted in accordance with the guidelines approved by the Institutional Animal Care and Use Committee of Sun Yat‐Sen University (Approval numbers: L102012020100N).

### Transcriptome Sequencing

Total RNA was extracted from 9 tumor tissues obtained from animal xenografts using the RNeasy Mini Kit (Qiagen, Germany). Paired‐end libraries were prepared using the TruSeq RNA Sample Preparation Kit (Illumina, USA) according to the manufacturer's instructions. First, mRNA with poly‐A tails was isolated using magnetic beads coated with poly‐T oligonucleotides. The purified mRNA was then fragmented into small pieces using divalent cations at 94 °C for 8 min. These RNA fragments were reverse transcribed into first‐strand cDNA using reverse transcriptase and random primers. Second‐strand cDNA synthesis was performed using DNA Polymerase I and RNase H. These cDNA fragments underwent end repair, the addition of a single ‘A’ base, and ligation of adapters. After purification and PCR enrichment, the final cDNA libraries were generated. The libraries were quantified using a Qubit 2.0 Fluorometer (Life Technologies, USA) and checked for insert size and concentration using an Agilent 2100 Bioanalyzer (Agilent Technologies, USA). Clustering was performed on a cBot system with the libraries diluted to 10 pm, followed by high‐throughput sequencing on the Illumina NovaSeq 6000 platform (Illumina, USA). Library construction and sequencing were conducted by Sinotech Genomics Co., Ltd (Shanghai, China).

### Data Processing

Paired‐end sequence files (FASTQ) were aligned to the reference genome using HISAT2 (Hierarchical Indexing for Spliced Alignment of Transcripts, version 2.0.5). The resulting SAM (Sequence Alignment/Map) files were converted to BAM (Binary Alignment/Map) format and sorted using SAMtools (version 1.3.1). Gene expression levels were quantified as fragments per kilobase of exon per million reads mapped (FPKM). StringTie software was utilized to count fragments mapped to each gene, and the normalization was performed using the TMM algorithm.

### Differential Gene Expression Analysis

Differential expression analysis of mRNA was conducted using the R package “edgeR”. Genes with a |log2(fold change) | greater than 1.5 and a q‐value less than 0.05 were considered significantly differentially expressed and selected for further analysis.

### Gene Set Enrichment Analysis

Gene set enrichment analysis (GSEA) was performed using the GSEA software^[^ ^65^ ^]^, which was downloaded from the Broad Institute website.^[^ ^66^ ^]^ The analysis was performed using the KEGG pathway database (c2.cp.kegg legacy.v2023.2.Hs.symbols.gmt). Pathways with a *p* < 0.05 were considered significantly enriched.

### Whole‐Tumor Immunolabeling and Optical Clearing

The tumor tissue‐clearing procedure was in accordance with advanced CUBIC methods developed by Matsumoto et al.^[^ ^67^ ^]^ Briefly, tumor samples were fixed in 4% paraformaldehyde on a shaker at 4 °C for 24 h. After being washed with PBS over 5 times, the samples were immersed in CUBIC‐L solution and incubated at 37 °C with shaking for 21 days. Following this, the CUBIC‐L solution was washed off with PBS more than 5 times, and the samples were sequentially stained with primary and secondary antibodies for a total of 14 days. Finally, the samples were incubated with CUBIC‐R solution to match the reflective index (RI) for subsequent imaging and data processing.

### 3D Imaging

Images were acquired using the BC43 multi‐function desktop confocal microscope (OXFORD Andor) at 10× magnification. A 3 × 3 montage was generated using Fusion. The imaging thickness was 250 µm with a step size of 2 µm.

### Statistical Analysis

All in vitro experiments were performed at least three times, and data are presented as the mean ± standard deviation (SD) from all replicates. Statistical analyses were conducted using GraphPad Prism 8 and R software (version 4.3.0). For comparisons between two groups, either an unpaired two‐tailed Student's *t*‐test or the Mann–Whitney *u* test was used, depending on data distribution. For comparisons among multiple groups, one‐way ANOVA followed by Tukey's post‐hoc test was applied. Categorical variables were analyzed using the Chi‐square test. Based on group sizes, either the Chi‐square test with Yates’ continuity correction or Fisher's exact test was used. To determine the optimal cut‐off value for TH and β2ARs expression in survival analysis, the “survminer” R package was used to identify thresholds that best separate patients with significantly different overall survival. Survival differences between groups were evaluated using the log‐rank test. A *p*‐value < 0.05 was considered statistically significant. Statistical significance is indicated as ^*^ *p* < 0.05, ^**^ *p* < 0.01, ^***^ *p* < 0.001 and ^****^ *p* < 0.0001. Details of statistical tests and sample sizes (*n*) are provided in the figure legends.

## Conflict of Interest

The authors declare no conflict of interest.

## Author Contributions

S.H., D.L., L.Y., and A.B. contributed equally to this work and shared first authorship. The project was conceived and supervised by Q.Q.C., Y.X., and M.N. The experiments were designed and performed by S.L.H., D.X.L., and M.N. Some experiments were performed by L.B.Y., A.W.B., Y.L.W, P.Z., D.Y.Z., and H.L.L. Data were independently extracted by H.L.L. and P.Z. according to predefined criteria. The quality of the included studies was independently evaluated by Y.L.W. and D.Y.Z. using the Newcastle Ottawa Scale. The patient samples for clinical data analysis were provided by Y.X. and M.N. The manuscript was prepared by S.L.H., D.X.L., and M.N. All authors read and approved the final manuscript.

## Supporting information



Supporting Information

## Data Availability

The datasets used and analyzed in this study are available from the corresponding author upon reasonable request.
