# Supplementary material for: Tumor‐Associated Sympathetic Nerves Promote the Progression of Epstein‐Barr Virus‐Positive Diffuse Large B‐Cell Lymphoma
Source: Adv Sci (Weinh). 2025 Jun 9;12(33):e13580. doi: 10.1002/advs.202413580 (PMC12412552; doi:10.1002/advs.202413580)
Supplement: Supplementary file 1 — Supporting Information [file ADVS-12-e13580-s001.docx]

***Supplementary materials for***

Tumor-Associated Sympathetic Nerves Promote the Progression of Epstein-Barr Virus-Positive Diffuse Large B-Cell Lymphoma

Silan Huang^1†^, Dexin Lei^1†^, Linbin Yang^2†^, Aiwei Bi^3†^, Yanlou Wang^1^, Peng Zhang^1^, Dongyu Zhuang^1^, Honglian Liu^1^, Qingqing Cai^1*^, Man Nie^1*^, Yi Xia^1*^

^1^Department of Medical Oncology, State Key Laboratory of Oncology in South China, Guangdong Provincial Clinical Research Center for Cancer, Collaborative Innovation Center for Cancer Medicine, Sun Yat-sen University Cancer Center, Guangzhou 510060, P. R. China.

^2^Breast Tumor Center, Guangdong Provincial Key Laboratory of Malignant Tumor Epigenetics and Gene Regulation, Medical Research Center, Sun Yat-sen Memorial Hospital, Sun Yat-sen University, Guangzhou, Guangdong, People’s Republic of China.

^3^Biotherapy Center, Sun Yat-sen Memorial Hospital, Sun Yat-sen University, Guangzhou 510120, China.

^†^ These authors have contributed equally to this work and share first authorship.

^*^ Shared corresponding authors. Correspondence and request for material should be addressed to Yi Xia ([xiayi@sysucc.org.cn](mailto:xiayi@sysucc.org.cn)), Man Nie (nieman@sysucc.org.cn) and Qingqing Cai (caiqq@sysucc.org.cn).

**This file includes:**

**Supplementary methods, supplementary figure 1-6 and supplementary Table 1-3.**

**Supplementary experimental methods**

**Systematic review of prognosis in EBV^+^ DLBCL versus DLBCL and the impact of beta-blocker use on cancer outcomes**

Meta-analysis of overall survival (OS) in EBV^+^ DLBCL *versus* DLBCL:

The search strategy used keywords such as “Epstein-Barr Virus,” “EB virus,” “EBV,” “human herpesvirus 4,” “HHV 4,” “diffuse large B-cell lymphoma,” and “DLBCL.”

**Inclusion criteria:** Clinical studies that compared the prognosis of patients with EBV^+^ DLBCL and DLBCL, reported overall survival (OS), and clearly described how DLBCL was diagnosed and determined the EBV status via in-situ hybridization.

**Exclusion criteria:** Studies with missing data, available only as abstracts, published in languages other than English, or based on animal or laboratory research.

Meta-analysis of the impact of beta-blocker usage on cancer outcomes:

The search strategy used a combination of keywords, including “beta-blocker”, “breast cancer”, “colorectal cancer”, “prostate cancer”, “pancreatic cancer”, and “usage”.

**Inclusion criteria:** Studies involving patients with breast, prostate, colorectal, or pancreatic cancers; cohort studies, case-control studies, or randomized controlled trials (including retrospective analyses of RCTs); studies that examined beta-blocker use as the exposure of interest (no minimum dose required); reported cancer-specific mortality as an outcome; and provided hazard ratio with 95% confidence intervals.

**Exclusion criteria:** Systematic reviews, laboratory experiments, case reports, ecological studies, and conference abstracts.

**Supplementary figures**

**Figure legend**

**Figure S1.** Establishment of Epstein-Barr virus (EBV)-infected diffuse large-B cell lymphoma (DLBCL) cell lines. (A) Relative expression of latency genes in xenograft tumors derived from SUDHL4-EBV cells. (B) Representative images of EBERs-ISH in tumor tissues from mice injected with SUDHL4-EBV cells.

**Figure S2.** Comparison of the biological behavior of EBV-infected cells *in vitro* and *in vivo*. (A-F) Equal numbers of SUDHL4 and SUDHL4-EBV cells were subcutaneously injected near the inguinal lymph node of NOD/SCID mice. Tumor growth was then monitored and compared, *n*=5 mice per group. Within one month after injection, SUDHL4-EBV tumors rapidly progressed and approached the ethical endpoint, whereas the parental SUDHL4 cells failed to form any palpable tumors during the same period and remained undetectable. (A) Tumor growth curves in NOD/SCID mice injected with either SUDHL4-EBV cells or SUDHL4 cells. (B-F) Images of tumor xenografts (B), tumor volumes (C) and tumor weights (D) of the at the time of sacrifice. Data are shown as mean ± SD. Student’s *t* test, ***P* < 0.01, *****P* < 0.0001. (E) Representative H&E and Ki-67 IHC staining images of tumor tissues from the indicated group. Scale bar=100 µm. (F) Quantification of Ki-67 staining in tumor sections. Data were analyzed using Student’s *t* test, *****P* < 0.0001.

**Figure S3.** The infiltration of nerve fibers in DLBCL. (A) Representative images of IF staining for nerve markers in tumor tissues from the indicated groups. Red arrows indicate nerve fibers. (B) Schematic illustration of the co-culture system using primary dorsal root ganglia (DRGs) and lymphoma cell lines. Created using Adobe Illustrator. (C) Representative images of DRGs stained with β-III tubulin after 3 days of co-culture with Farage cells. Color-coded by penetration depth. (D) Quantification of neurite growth, shown as a proportion of the field covered by neurites in DRG co-cultures. Data was obtained from three independent experiments. Statistical analysis was performed using Student’s t test, **P* < 0.05.

**Figure S4.** Sympathetic nerves promote the growth of EBV^+^ DLBCL tumors in vivo. (A-G) NOD/SCID mice bearing SUDHL4-EBV tumors were treated with either PBS or 6-OHDA and sacrificed on day 21 post-inoculation. *n*=5 mice/group. (A) Schematic diagram showing 6-OHDA-induced peripheral sympathectomy. (B-C) Representative immunofluorescent images (B) and quantification (C) of sympathetic nerve fibers in tumors from the indicated group. Red arrows indicate nerve fibers. TH (green), NF-H (red), DAPI (blue). Scale bar: 10 μm. Data were analyzed using the Mann Whitney *u* test. ***P* < 0.01. (D) Tumor growth curve of SUDHL4-EBV xenografts in the PBS and 6-OHDA treated groups. (E-F) Tumor volume (E) and tumor weight (F) on day 21. Data analyzed using Student’s *t* test. **P* < 0.05. (G) The images of tumor xenografts on day 21. (H) Experimental scheme showing the treatment of SUDHL6-EBV tumor-bearing mice with daily intraperitoneal injections of the non-selective muscarinic receptor antagonist scopolamine (SCO). PBS was used as a control. (I) Tumor growth curve of SUDHL6-EBV xenografts in SCO and PBS treated group. Student’s t test, *ns*: not significant. *n*=5 mice/group.

**Figure S5.** Sympathetic nerves promote the growth of EBV^+^ DLBCL via β2 adrenergic receptor. (A-G) NOD/SCID mice bearing SUDHL4-EBV tumors were treated daily with PBS, the selective β2-adrenergic receptor antagonist ICI-118551 (ICI) or the selective β3-adrenergic receptor antagonist SR59230A (SR) and were sacrificed on day 18 post-inoculation. *n*=6 mice/group. (A) Experimental scheme showing the treatment of SUDHL4-EBV tumor-bearing mice with daily injections of ICI, SR, or PBS as control. (B) Tumor growth curves for the different treatment groups. (C-D) Tumor volume (C) and tumor weight (D) on day 18. Data were analyzed by one-way ANOVA. **P* < 0.05, ***P* < 0.01, *ns*: not significant. (E) Images of tumor xenografts on day 18. (F-G) Representative immunofluorescent images (F) and quantification (G) of sympathetic nerve fibers in tumors from each group. Red arrows indicate nerve fibers. TH (green), NF-H (red), DAPI (blue). Scale bar: 10 μm. Data were analyzed using the Kruskal Wallis test. *ns*: not significant.

**Figure S6.** Forest plots showing the association between beta-blocker usage and cancer specific mortality.

**Supplementary figures**


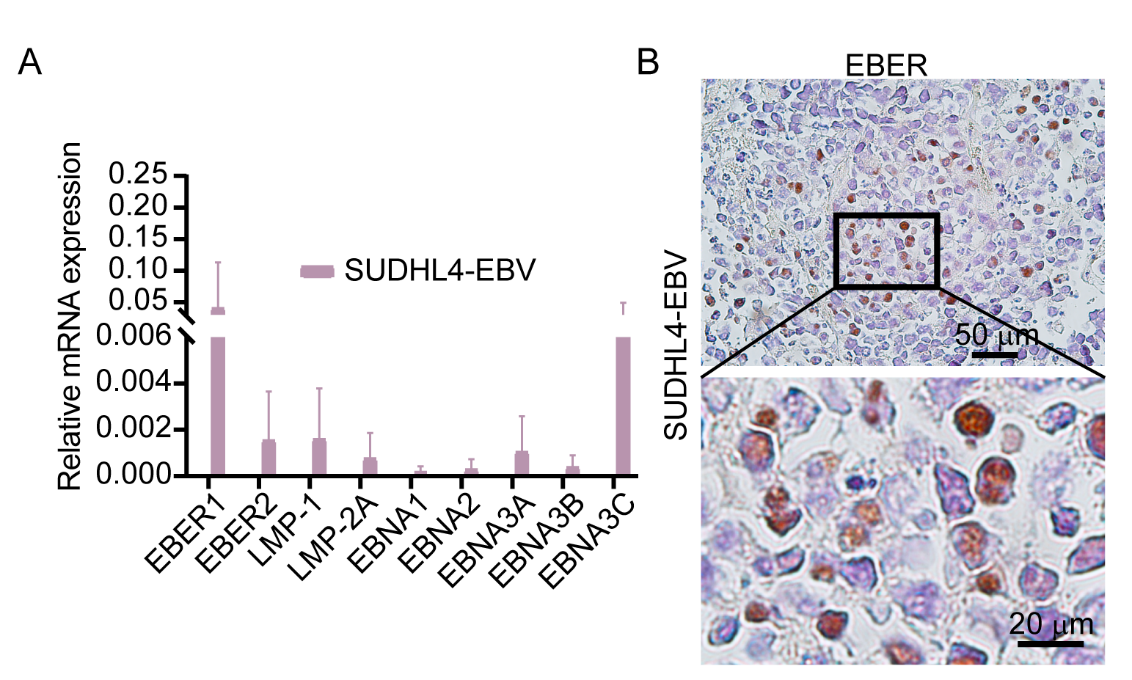


Figure S1


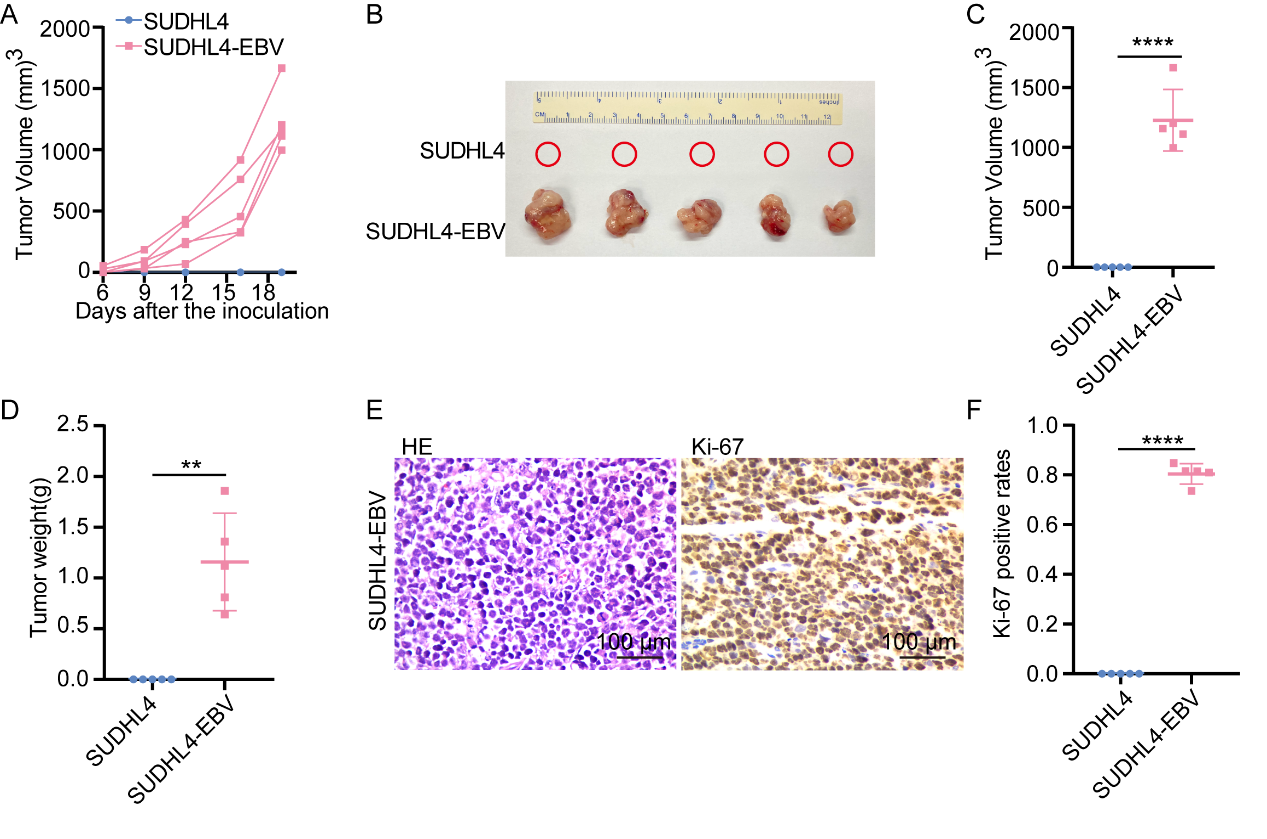


Figure S2


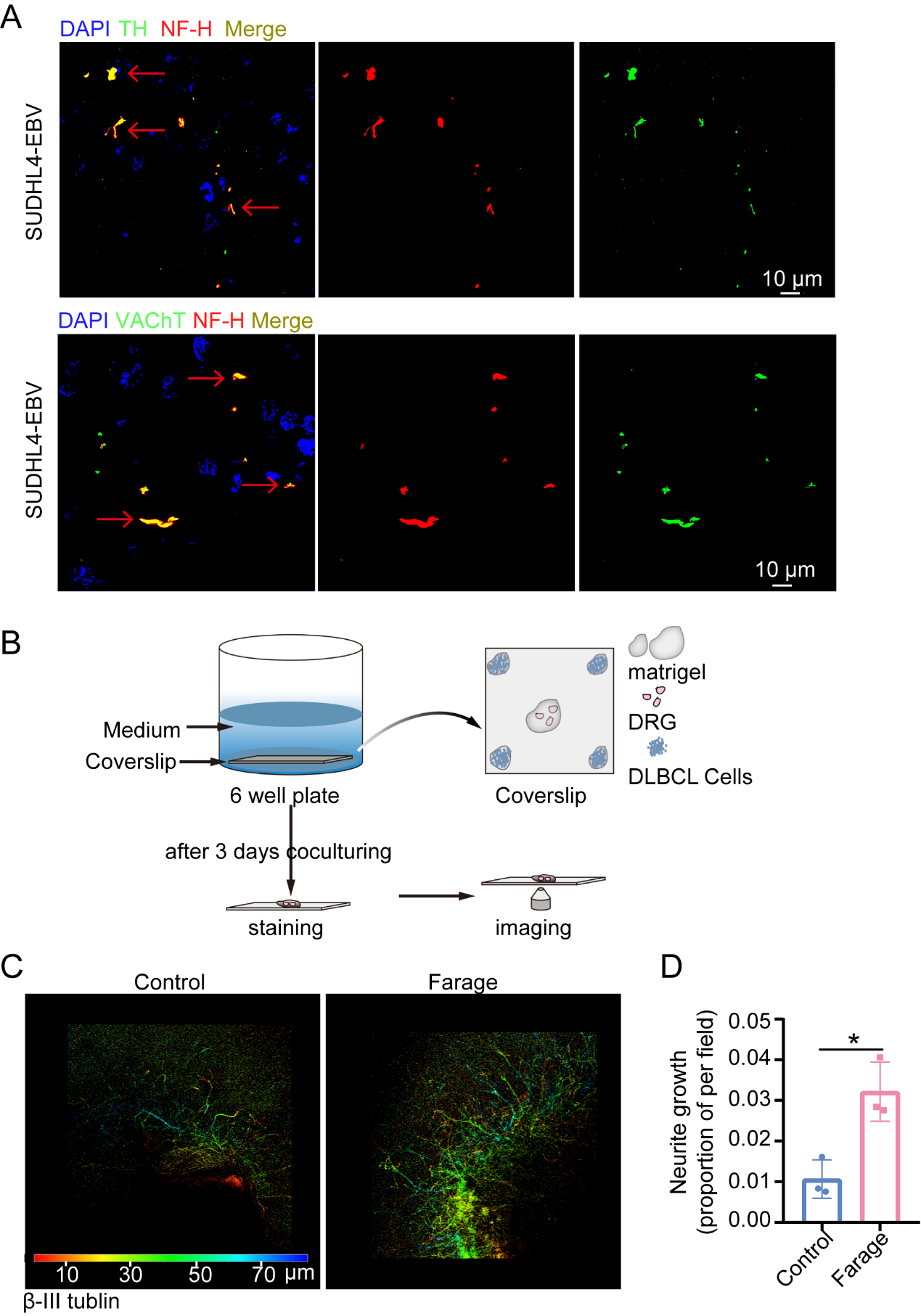


Figure S3


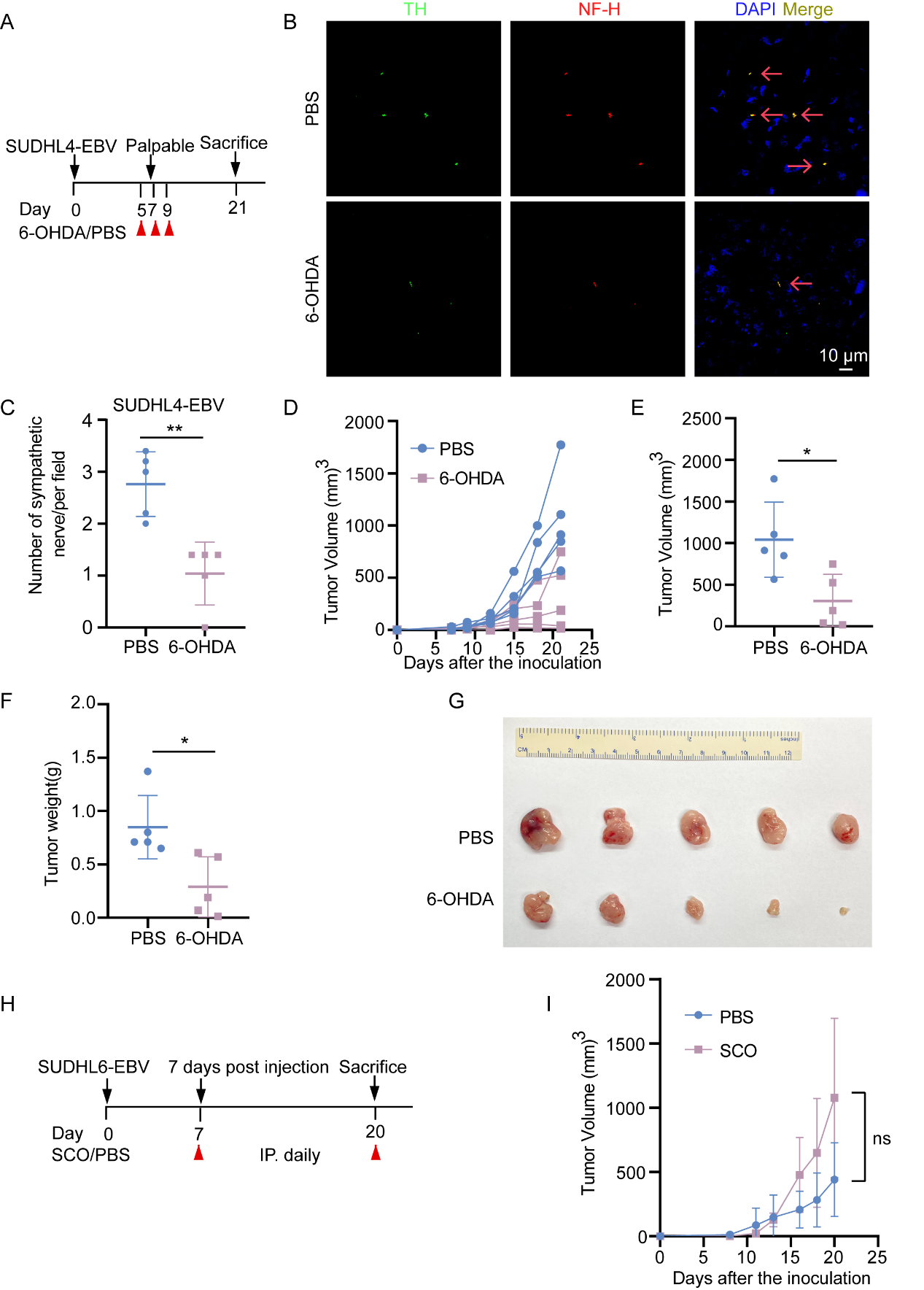


Figure S4


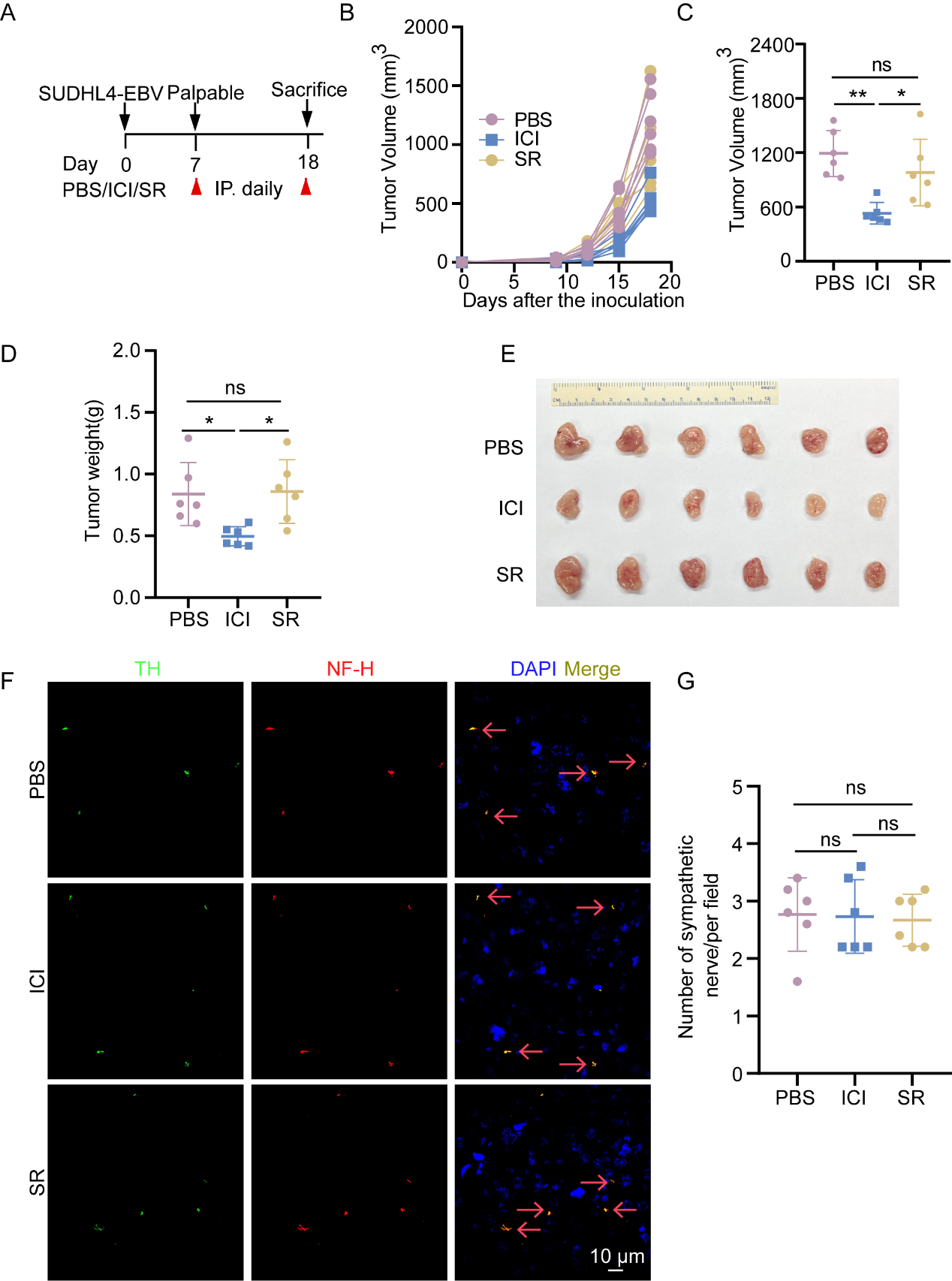


Figure S5


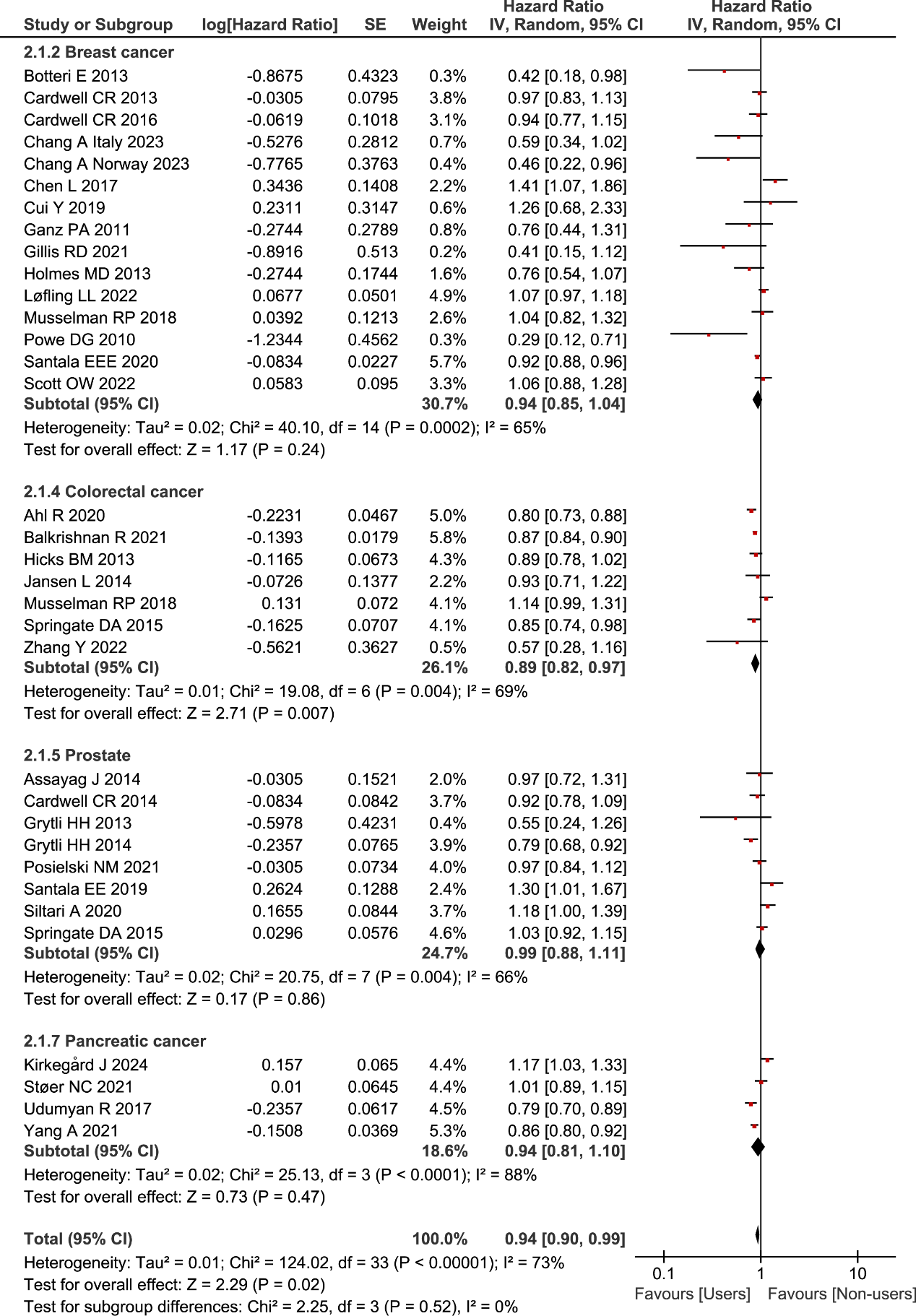


Figure S6

**Supplementary Tables**

Supplementary Table 1: Primer of qPCR

EBER1 Forward 5'-GAGGTTTTGCTAGGGAGGAGAC-3'

Reverse 5'-GAAGACGGCAGAAAGCAGAGT-3'

EBER2 Forward 5'-AACGCTCAGTGCGGTGCTA-3'

Reverse 5'- GCCGAATACCCTTCTCCCA-3'

LMP1 Forward 5'-GTATTGGCACAAGATGGAAAGC-3'

Reverse 5'-CAACTACCAGGCAGATGAGGC-3'

LMP2A Forward 5'-ACGATGGCGGAAACAACTC-3'

Reverse 5'-GGGTCCTCATAAGGCGGTG-3'

EBNA1 Forward 5'-GTAGGGGATGCCGATTATTTTG-3'

Reverse 5'-CTCCTTGACCACGATGCTTTC-3'

EBNA2 Forward 5'-CATCTGCTATGCGAATGCTT-3'

Reverse 5'-ATGTGGCTGGACCAACCTG-3'

EBNA3A Forward 5'-GTTTTTAGCGACGGGCGAGTG-3'

Reverse 5'-GACAGGGACGGGTTCTACT-3'

EBNA3B Forward 5'-CATCCTGTCGCCATTCCTTAT-3'

Reverse 5'-CCATTCCACTCTTGTCTTCCTCC-3'

EBNA3C Forward 5'-CATTAACTCCCGTGCCTAACAG-3'

Reverse 5'-TCCCGAGCGTATCTTTCTTGC-3'

β-actin Forward 5'-AGAGCTACGAGCTGCCTGAC-3'

Reverse 5'-AGCACTGTGTTGGCGTACAG-3'

Supplementary Table 2: Main characteristics and the Newcastle-Ottawa Scale score of the eligible studies included in meta-analysis of EBV^+^DLBCL *versus* DLBCL.

| First author | Year | Country | Median follow-up (months) | Sample size (EBV^+^ DLBCL/DLBCL,*n*) | Data extraction | Newcastle-Ottawa Scale score | | | |
| --- | --- | --- | --- | --- | --- | --- | --- | --- | --- |
|  |  |  |  |  |  | selection | comparability | outcome | total score |
| Kuze T^1^ | 2000 | Japan | NR^*^ | 12/58 | KM^*^ | 3 | 0 | 3 | 6 |
| Park S^2^ | 2007 | Korea | 40.5 | 34/346 | KM | 4 | 0 | 3 | 7 |
| Morales D^3^ | 2010 | Peru | NR | 6/51 | Direct | 4 | 0 | 3 | 7 |
| Ahn JS^4^ | 2013 | Korea | 32.8 | 18/204 | KM | 4 | 0 | 3 | 7 |
| Sato A^5^ | 2014 | Japan | 25.2 | 14/225 | Direct | 4 | 1 | 3 | 8 |
| Ok CY^6^ | 2014 | USA | 42.1 | 28/675 | Direct | 4 | 1 | 3 | 8 |
| Lu CH^7^ | 2014 | Taiwan, China | NR | 15/74 | KM | 4 | 0 | 3 | 7 |
| Chang ST^8^ | 2014 | Taiwan, China | 14 | 15/317 | Direct | 4 | 1 | 3 | 8 |
| Lu TX^9^ | 2015 | China | 29.3 | 35/215 | KM | 4 | 0 | 3 | 7 |
| Hong JY^10^ | 2015 | Korea | 102.5 | 48/523 | Direct | 4 | 1 | 3 | 8 |
| Liang JH^11^ | 2015 | China | 38 | 24/208 | Direct | 4 | 1 | 3 | 8 |
| Okamoto A^12^ | 2015 | Japan | 40 | 11/123 | KM | 4 | 0 | 3 | 7 |
| Chuang WY^13^ | 2015 | Taiwan, China | NR | 10/164 | Direct | 4 | 1 | 3 | 8 |
| Song CG^14^ | 2015 | China | 47 | 16/48 | KM | 4 | 1 | 3 | 8 |
| Hong JY^15^ | 2017 | Korea | 42.2 | 14/196 | Direct | 4 | 1 | 3 | 8 |
| Beltran BE^16^ | 2018 | USA | NR | 17/84 | Direct | 4 | 0 | 3 | 7 |
| Tracy SI^17^ | 2018 | USA | 59 | 16/346 | Direct | 4 | 1 | 3 | 8 |
| Keane C^18^ | 2019 | Australia | 45 | 30/353 | KM | 4 | 0 | 3 | 7 |
| Witte HM^19^ | 2020 | Germany | 42 | 80/76 | KM | 4 | 0 | 3 | 7 |
| Bourbon E^20^ | 2021 | Franc | 48 | 56/425 | KM | 4 | 0 | 3 | 7 |
| Shen Z^21^ | 2022 | China | 28.6 | 5/90 | KM | 4 | 0 | 3 | 7 |

Supplementary Table 3:

Main characteristics and the Newcastle-Ottawa Scale score of the eligible studies included in meta-analysis the impact of beta-blocker usage on cancer outcomes.

| Cancer site | Author | Year | Country | Follow-up time | Sample size | Newcastle-Ottawa Scale score | | | |
| --- | --- | --- | --- | --- | --- | --- | --- | --- | --- |
|  |  |  |  |  |  | selection | comparability | outcome | total score |
| Prostate | Cardwell CR^22^ | 2014 | UK | 7Y | 4715 | 4 | 1 | 3 | 8 |
|  | Grytli HH^23^ | 2013 | Norway | 122 M | 6303 | 4 | 1 | 3 | 8 |
|  | Grytli HH^24^ | 2014 | Norway | 39M | 24571 | 4 | 1 | 3 | 8 |
|  | Posielski NM^25^ | 2021 | USA | 134M | 39198 | 4 | 1 | 3 | 8 |
|  | Santala EE^26^ | 2019 | Finland | 9.9Y | 14422 | 4 | 1 | 3 | 8 |
|  | Siltari A^27^ | 2020 | Finland | 7.6Y | 8253 | 4 | 1 | 3 | 8 |
|  | Springate DA^28^ | 2015 | UK | 29.5M | 3462 | 4 | 0 | 3 | 7 |
|  | Assayag J^29^ | 2014 | UK | 3.8Y | 6270 | 4 | 1 | 3 | 8 |
| Total |  |  |  |  | 107194 |  |  |  |  |
| breast cancer | Powe DG^30^ | 2010 | UK | 124M | 466 | 4 | 1 | 3 | 8 |
|  | Cardwell C^31^ | 2013 | UK | 6 Y | 7132 | 4 | 1 | 3 | 8 |
|  | Botteri E^32^ | 2013 | US | 71 M | 800 | 4 | 1 | 3 | 8 |
|  | Holmes MD^33^ | 2013 |  | 10.5Y | 4661 | 4 | 1 | 3 | 8 |
|  | Ganz PA^34^ | 2011 | European | 8.2 Y | 1779 | 4 | 0 | 3 | 7 |
|  | Cardwell CR^35^ | 2016 |  | 4-6 Y | 188503 | 4 | 1 | 3 | 8 |
|  | Chen L^36^ | 2017 |  | 3 Y | 14766 | 4 | 1 | 3 | 8 |
|  | Musselman RP^37^ | 2018 |  | 57.6M | 30020 | 4 | 0 | 3 | 7 |
|  | Chang A^38^ | 2023 | Italy | NR | 1135 | 4 | 1 | 3 | 8 |
|  |  |  | Norway | NR | 907 | 4 | 1 | 3 | 8 |
|  | Løfling LL^39^ | 2022 | Norway | 5.1 Y | 30060 | 4 | 1 | 3 | 8 |
|  | Scott OW^40^ | 2022 | New Zealand | 4.51 Y | 14976 | 4 | 1 | 3 | 8 |
|  | Gillis RD^41^ | 2021 | Norway | 5.5 Y | 4014 | 4 | 1 | 3 | 8 |
|  | Santala EEE^42^ | 2020 | Finland | 5.8-6.2 Y | 73170 | 4 | 1 | 3 | 8 |
|  | Cui Y^43^ | 2019 | China | 3.4 Y | 633 | 4 | 1 | 3 | 8 |
| Total |  |  |  |  | 373022 |  |  |  |  |
| pancreatic cancer. | Støer NC^44^ | 2021 | Norway | 6M | 2614 | 4 | 1 | 3 | 8 |
|  | Yang A^45^ | 2021 | US | NR | 13731 | 4 | 1 | 3 | 8 |
|  | Kirkegård J^46^ | 2024 | Danis | NR | 2592 | 4 | 1 | 3 | 8 |
|  | Udumyan R^47^ | 2017 | Sweden | 5M | 2394 | 4 | 1 | 3 | 8 |
| Total |  |  |  |  | 21331 |  |  |  |  |
| colorectal cancer | B M Hicks^48^ | 2013 | UK | 6.2Y | 4794 | 4 | 1 | 3 | 8 |
|  | Jansen L^49^ | 2014 | southwest Germany | 5Y | 1975 | 4 | 1 | 3 | 8 |
|  | Zhang Y^50^ | 2022 | US | Up to 28Y | 110431 | 4 | 0 | 3 | 7 |
|  | Balkrishnan R^51^ | 2021 | US | NR | 13982 | 4 | 1 | 3 | 8 |
|  | Ahl R^52^ | 2020 | Sweden | NR | 22337 | 4 | 1 | 3 | 8 |
|  | Springate DA^28^ | 2015 | UK | 29.5M | 3462 | 4 | 0 | 3 | 7 |
| Total |  |  |  |  | 156981 |  |  |  |  |
| All total |  |  |  |  | 658528 |  |  |  |  |

**Reference:**

1. Kuze T, Nakamura N, Hashimoto Y, Sasaki Y, Abe M. The characteristics of Epstein-Barr virus (EBV)-positive diffuse large B-cell lymphoma: comparison between EBV(+) and EBV(-) cases in Japanese population. Japanese journal of cancer research : Gann 2000;91:1233-40.

2. Park S, Lee J, Ko YH, et al. The impact of Epstein-Barr virus status on clinical outcome in diffuse large B-cell lymphoma. Blood 2007;110:972-8.

3. Morales D, Beltran B, De Mendoza FH, et al. Epstein-Barr virus as a prognostic factor in de novo nodal diffuse large B-cell lymphoma. Leuk Lymphoma 2010;51:66-72.

4. Ahn JS, Yang DH, Duk Choi Y, et al. Clinical outcome of elderly patients with Epstein-Barr virus positive diffuse large B-cell lymphoma treated with a combination of rituximab and CHOP chemotherapy. Am J Hematol 2013;88:774-9.

5. Sato A, Nakamura N, Kojima M, et al. Clinical outcome of Epstein-Barr virus-positive diffuse large B-cell lymphoma of the elderly in the rituximab era. Cancer Sci 2014;105:1170-5.

6. Ok CY, Li L, Xu-Monette ZY, et al. Prevalence and clinical implications of epstein-barr virus infection in de novo diffuse large B-cell lymphoma in Western countries. Clin Cancer Res 2014;20:2338-49.

7. Lu CH, Lee KF, Chen CC, et al. Clinical characteristics and treatment outcome in a Taiwanese population of patients with Epstein-Barr virus-positive diffuse large B-cell lymphoma. Jpn J Clin Oncol 2014;44:1164-71.

8. Chang ST, Lu YH, Lu CL, et al. Epstein-Barr virus is rarely associated with diffuse large B cell lymphoma in Taiwan and carries a trend for a shorter median survival time. Journal of clinical pathology 2014;67:326-32.

9. Lu TX, Liang JH, Miao Y, et al. Epstein-Barr virus positive diffuse large B-cell lymphoma predict poor outcome, regardless of the age. Sci Rep 2015;5:12168.

10. Hong JY, Yoon DH, Suh C, et al. EBV-positive diffuse large B-cell lymphoma in young adults: is this a distinct disease entity? Annals of oncology : official journal of the European Society for Medical Oncology 2015;26:548-55.

11. Liang JH, Lu TX, Tian T, et al. Epstein-Barr virus (EBV) DNA in whole blood as a superior prognostic and monitoring factor than EBV-encoded small RNA in situ hybridization in diffuse large B-cell lymphoma. Clinical microbiology and infection : the official publication of the European Society of Clinical Microbiology and Infectious Diseases 2015;21:596-602.

12. Okamoto A, Yanada M, Inaguma Y, et al. The prognostic significance of EBV DNA load and EBER status in diagnostic specimens from diffuse large B-cell lymphoma patients. Hematol Oncol 2017;35:87-93.

13. Chuang WY, Chang H, Shih LY, et al. CD5 positivity is an independent adverse prognostic factor in elderly patients with diffuse large B cell lymphoma. Virchows Archiv : an international journal of pathology 2015;467:571-82.

14. Song CG, Huang JJ, Li YJ, et al. Epstein-Barr Virus-Positive Diffuse Large B-Cell Lymphoma in the Elderly: A Matched Case-Control Analysis. PLoS One 2015;10:e0133973.

15. Hong JY, Ryu KJ, Park C, et al. Clinical impact of serum survivin positivity and tissue expression of EBV-encoded RNA in diffuse large B-cell lymphoma patients treated with rituximab-CHOP. Oncotarget 2017;8:13782-91.

16. Beltran BE, Quiñones P, Morales D, et al. Response and survival benefit with chemoimmunotherapy in Epstein-Barr virus-positive diffuse large B-cell lymphoma. Hematol Oncol 2018;36:93-7.

17. Tracy SI, Habermann TM, Feldman AL, et al. Outcomes among North American patients with diffuse large B-cell lymphoma are independent of tumor Epstein-Barr virus positivity or immunosuppression. Haematologica 2018;103:297-303.

18. Keane C, Tobin J, Gunawardana J, et al. The tumour microenvironment is immuno-tolerogenic and a principal determinant of patient outcome in EBV-positive diffuse large B-cell lymphoma. European journal of haematology 2019;103:200-7.

19. Witte HM, Merz H, Biersack H, et al. Impact of treatment variability and clinicopathological characteristics on survival in patients with Epstein-Barr-Virus positive diffuse large B cell lymphoma. British journal of haematology 2020;189:257-68.

20. Bourbon E, Maucort-Boulch D, Fontaine J, et al. Clinicopathological features and survival in EBV-positive diffuse large B-cell lymphoma not otherwise specified. Blood Adv 2021;5:3227-39.

21. Shen Z, Hu L, Yao M, et al. Disparity analysis and prognostic value of pretreatment whole blood Epstein-Barr virus DNA load and Epstein-Barr encoding region status in lymphomas: A retrospective multicenter study in Huaihai Lymphoma Working Group. International journal of cancer 2022;150:327-34.

22. Cardwell CR, Coleman HG, Murray LJ, O'Sullivan JM, Powe DG. Beta-blocker usage and prostate cancer survival: a nested case-control study in the UK Clinical Practice Research Datalink cohort. Cancer epidemiology 2014;38:279-85.

23. Grytli HH, Fagerland MW, Fosså SD, Taskén KA, Håheim LL. Use of β-blockers is associated with prostate cancer-specific survival in prostate cancer patients on androgen deprivation therapy. The Prostate 2013;73:250-60.

24. Grytli HH, Fagerland MW, Fosså SD, Taskén KA. Association between use of β-blockers and prostate cancer-specific survival: a cohort study of 3561 prostate cancer patients with high-risk or metastatic disease. European urology 2014;65:635-41.

25. Posielski NM, Richards KA, Liou JI, et al. Beta-Adrenergic Antagonists and Cancer Specific Survival in Patients With Advanced Prostate Cancer: A Veterans Administration Cohort Study. Urology 2021;155:186-91.

26. Santala EE, Rannikko A, Murtola TJ. Antihypertensive drugs and prostate cancer survival after radical prostatectomy in Finland-A nationwide cohort study. International journal of cancer 2019;144:440-7.

27. Siltari A, Murtola TJ, Talala K, Taari K, Tammela TLJ, Auvinen A. Antihypertensive drug use and prostate cancer-specific mortality in Finnish men. PLoS One 2020;15:e0234269.

28. Springate DA, Ashcroft DM, Kontopantelis E, Doran T, Ryan R, Reeves D. Can analyses of electronic patient records be independently and externally validated? Study 2--the effect of β-adrenoceptor blocker therapy on cancer survival: a retrospective cohort study. BMJ open 2015;5:e007299.

29. Assayag J, Pollak MN, Azoulay L. Post-diagnostic use of beta-blockers and the risk of death in patients with prostate cancer. European journal of cancer (Oxford, England : 1990) 2014;50:2838-45.

30. Powe DG, Voss MJ, Zänker KS, et al. Beta-blocker drug therapy reduces secondary cancer formation in breast cancer and improves cancer specific survival. Oncotarget 2010;1:628-38.

31. Cardwell CR, Coleman HG, Murray LJ, Entschladen F, Powe DG. Beta-blocker usage and breast cancer survival: a nested case-control study within a UK clinical practice research datalink cohort. International journal of epidemiology 2013;42:1852-61.

32. Botteri E, Munzone E, Rotmensz N, et al. Therapeutic effect of β-blockers in triple-negative breast cancer postmenopausal women. Breast cancer research and treatment 2013;140:567-75.

33. Holmes MD, Hankinson SE, Feskanich D, Chen WY. Beta blockers and angiotensin-converting enzyme inhibitors' purported benefit on breast cancer survival may be explained by aspirin use. Breast cancer research and treatment 2013;139:507-13.

34. Ganz PA, Habel LA, Weltzien EK, Caan BJ, Cole SW. Examining the influence of beta blockers and ACE inhibitors on the risk for breast cancer recurrence: results from the LACE cohort. Breast cancer research and treatment 2011;129:549-56.

35. Cardwell CR, Pottegård A, Vaes E, et al. Propranolol and survival from breast cancer: a pooled analysis of European breast cancer cohorts. Breast cancer research : BCR 2016;18:119.

36. Chen L, Chubak J, Boudreau DM, Barlow WE, Weiss NS, Li CI. Use of Antihypertensive Medications and Risk of Adverse Breast Cancer Outcomes in a SEER-Medicare Population. Cancer epidemiology, biomarkers & prevention : a publication of the American Association for Cancer Research, cosponsored by the American Society of Preventive Oncology 2017;26:1603-10.

37. Musselman RP, Bennett S, Li W, et al. Association between perioperative beta blocker use and cancer survival following surgical resection. European journal of surgical oncology : the journal of the European Society of Surgical Oncology and the British Association of Surgical Oncology 2018;44:1164-9.

38. Chang A, Botteri E, Gillis RD, et al. Beta-blockade enhances anthracycline control of metastasis in triple-negative breast cancer. Sci Transl Med 2023;15:eadf1147.

39. Løfling LL, Støer NC, Sloan EK, et al. β-blockers and breast cancer survival by molecular subtypes: a population-based cohort study and meta-analysis. British journal of cancer 2022;127:1086-96.

40. Scott OW, Tin Tin S, Elwood JM, et al. Post-diagnostic beta blocker use and breast cancer-specific mortality: a population-based cohort study. Breast cancer research and treatment 2022;193:225-35.

41. Gillis RD, Botteri E, Chang A, et al. Carvedilol blocks neural regulation of breast cancer progression in vivo and is associated with reduced breast cancer mortality in patients. European journal of cancer (Oxford, England : 1990) 2021;147:106-16.

42. Santala EEE, Murto MO, Artama M, Pukkala E, Visvanathan K, Murtola TJ. Angiotensin Receptor Blockers Associated with Improved Breast Cancer Survival-A Nationwide Cohort Study from Finland. Cancer epidemiology, biomarkers & prevention : a publication of the American Association for Cancer Research, cosponsored by the American Society of Preventive Oncology 2020;29:2376-82.

43. Cui Y, Wen W, Zheng T, et al. Use of Antihypertensive Medications and Survival Rates for Breast, Colorectal, Lung, or Stomach Cancer. American journal of epidemiology 2019;188:1512-28.

44. Støer NC, Bouche G, Pantziarka P, Sloan EK, Andreassen BK, Botteri E. Use of non-cancer drugs and survival among patients with pancreatic adenocarcinoma: a nationwide registry-based study in Norway. Acta oncologica (Stockholm, Sweden) 2021;60:1146-53.

45. Yang A, Zylberberg HM, Rustgi SD, et al. Beta-blockers have no impact on survival in pancreatic ductal adenocarcinoma prior to cancer diagnosis. Sci Rep 2021;11:1038.

46. Kirkegård J, Cronin-Fenton D, Lund A, Mortensen FV. Beta-blocker use and survival after pancreatic cancer surgery: A nationwide population-based cohort study. Pharmacoepidemiology and drug safety 2024;33:e5726.

47. Udumyan R, Montgomery S, Fang F, et al. Beta-Blocker Drug Use and Survival among Patients with Pancreatic Adenocarcinoma. Cancer Res 2017;77:3700-7.

48. Hicks BM, Murray LJ, Powe DG, Hughes CM, Cardwell CR. β-Blocker usage and colorectal cancer mortality: a nested case-control study in the UK Clinical Practice Research Datalink cohort. Annals of oncology : official journal of the European Society for Medical Oncology 2013;24:3100-6.

49. Jansen L, Hoffmeister M, Arndt V, Chang-Claude J, Brenner H. Stage-specific associations between beta blocker use and prognosis after colorectal cancer. Cancer 2014;120:1178-86.

50. Zhang Y, Song M, Chan AT, Meyerhardt JA, Willett WC, Giovannucci EL. Long-term use of antihypertensive medications, hypertension and colorectal cancer risk and mortality: a prospective cohort study. British journal of cancer 2022;127:1974-82.

51. Balkrishnan R, Desai RP, Narayan A, Camacho FT, Flausino LE, Chammas R. Associations between initiating antihypertensive regimens on stage I-III colorectal cancer outcomes: A Medicare SEER cohort analysis. Cancer Med 2021;10:5347-57.

52. Ahl R, Matthiessen P, Sjölin G, et al. Effects of beta-blocker therapy on mortality after elective colon cancer surgery: a Swedish nationwide cohort study. BMJ open 2020;10:e036164.
